# CypD Dependent mPTP Opening Is Crucial for Oxidized Mitochondrial DNA Release in Ferroptosis

**DOI:** 10.1002/advs.202502239

**Published:** 2026-02-17

**Authors:** Hong Zhou, Wan Fu, Shizuo Liu, Zili Zhang, Hanyan Luo, Qing Zhong

**Affiliations:** ^1^ Institute For Translational Medicine On Cell Fate and Disease Department of Pathophysiology Shanghai Ninth People's Hospital Key Laboratory of Cell Differentiation and Apoptosis of Chinese Ministry of Education Shanghai Jiao Tong University School of Medicine Shanghai China

**Keywords:** cGAS‐STING, ferroptosis, mitochondria, mPTP, mtDNA

## Abstract

Ferroptosis is a type of regulated cell death characterized by the accumulation of lipid peroxides that damage cell membranes specifically. Mitochondrial swelling and dysfunction are hallmarks of ferroptosis; however, what causes mitochondrial swelling and the consequences of mitochondrial swelling in ferroptotic signal transduction remain poorly understood. Our study found that mitochondrial permeability transition pore (mPTP) opening is essential for mitochondrial swelling and ferroptosis activation. During ferroptosis, oxidized mitochondrial DNAs (mtDNAs) are released through the mPTP. These oxidized mtDNAs activate the cyclic GMP‐AMP synthase (cGAS)—stimulator of interferon genes (STING) pathway, promoting ferroptosis through activating ferrotinophagy. Consistently, inhibition of mtDNA‐repair enhances cellular sensitivity to ferroptosis and therefore synergizes with ferroptosis inducer in suppressing tumorigenesis in mouse xenograft tumor models. This study provides a fundamental understanding of how mPTP engages in ferroptosis by releasing mitochondrial DNAs as crucial messengers to activate ferroptotic signaling.

## Graphical abstract

Ferroptosis is driven by mitochondrial permeability transition pore (mPTP) opening, which induces mitochondrial swelling and releases oxidized mitochondrial DNA. The released mtDNA activates cGAS–STING signaling, promotes ferritinophagy, and amplifies ferroptotic cell death. Disruption of mtDNA repair sensitizes tumors to ferroptosis in vivo, revealing a mitochondria‐initiated signaling axis in ferroptosis regulation.

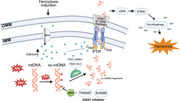

## 1 Introduction

Ferroptosis is a unique form of programmed cell death characterized by iron‐dependent lipid peroxidation [1, 2, 3]. Unlike apoptosis or necrosis, ferroptosis is driven by the accumulation of lipid peroxides due to increased intracellular iron [1]. This iron catalyzes the production of reactive oxygen species (ROS) through Fenton reactions, leading to the oxidation of polyunsaturated fatty acids (PUFAs) in cell membranes [2, 4, 5, 6]. The resulting lipid peroxidation compromises membrane integrity, ultimately causing cell death [2, 3, 7]. While lipid peroxidation is recognized to be a hallmark of ferroptosis, it still remains unknown whether other oxidized substrates, like proteins or nucleic acid exhibit regulatory functions in ferroptosis.

Mitochondrion is a fundamental organelle for cell survival due to the essential mitochondrial oxidative phosphorylation system (OXPHOS) for energy production [8]. Dysfunction of mitochondria is involved in cell death scenario including apoptosis, necroptosis, and pyroptosis [9, 10, 11, 12]. The relationship between mitochondria and ferroptosis is complex [13]. Mitochondria are key players in the initiation and execution of ferroptosis due to their roles in ROS production, iron metabolism, and lipid peroxidation [7, 14]. Mitochondria‐resident dihydroorotate dehydrogenase (DHODH) and glutathione peroxidase 4 (GPX4) constitute two major defensive arms to detoxify lipid peroxides, which lead to ferroptosis [15, 16]. Moreover, mitochondria also provide essential precursors and conditions for glutathione production and import, which is a key antioxidant that protects cells from oxidative damage [17, 18]. Nevertheless, it is unsolved whether mitochondria function as a ROS‐unrelated signal transducer during the ferroptosis process.

mPTP is a non‐specific channel that forms across the mitochondrial membranes under certain stress conditions [19]. mPTP senses multiple cellular factors like calcium overload, ROS elevation, energy depletion, and mitochondrial membrane potential (Δψm) collapse [19, 20, 21]. When the mPTP opens, it disrupts the mitochondrial membrane potential, leading to the loss of ATP production, swelling of the mitochondria, and release of small molecules (e.g., cytochrome c, mtDNA) into the cytosol [19, 21, 22, 23]. This cascade of events can lead to necrosis or apoptosis, depending on the extent of mitochondrial damage [24]. mPTP‐driven cell death is implicated in various pathological conditions, including ischemia‐reperfusion injury, neurodegeneration, and heart disease [25, 26, 27, 28]. Our recent study demonstrated that during ferroptosis, MAMs mediated calcium transfer leads to mitochondrial calcium overload and thereby enhances ROS production and lipid peroxidation in mitochondria [29]. However, whether mPTP is involved and plays a significant role in ferroptosis has yet to be investigated.

The cyclic GMP‐AMP synthase‐Stimulator of Interferon Genes (cGAS‐STING) pathway plays a crucial role in the immune system [30]. cGAS acts as a cytosolic DNA sensor [31, 32, 33, 34, 35]. Upon detecting foreign or aberrant DNA within the cell, cGAS is activated and synthesizes cyclic GMP‐AMP (cGAMP). cGAMP then binds to the STING protein, leading to its conformational change and activation of downstream IRF3‐NF‐κB signaling pathways, initiating antiviral and antitumor immune responses [32, 33, 34, 35, 36, 37]. cGAS‐STING activation has been found in multiple cell death pathways, including apoptosis, pyroptosis, and ferroptosis [38, 39, 40, 41, 42]. The molecular mechanism igniting cGAS‐STING during ferroptosis remains unelucidated.

In this study, through chemical and genetic studies, we found that mPTPs enhanced ferroptosis by mediating the release of oxidized mitochondrial DNA to the cytosol. The released mtDNA then activates the cGAS‐STING signaling pathway, leading to an increase in ferrotinophagy mediated by labile iron and execution of ferroptosis. The mPTP‐mtDNA‐cGAS‐STING axis is a crucial regulatory signaling pathway in ferroptosis.

## 2 Results

### 2.1 Knock Out of Cyclophilin D (CypD) Protects Ferroptosis

**FIGURE 1:**
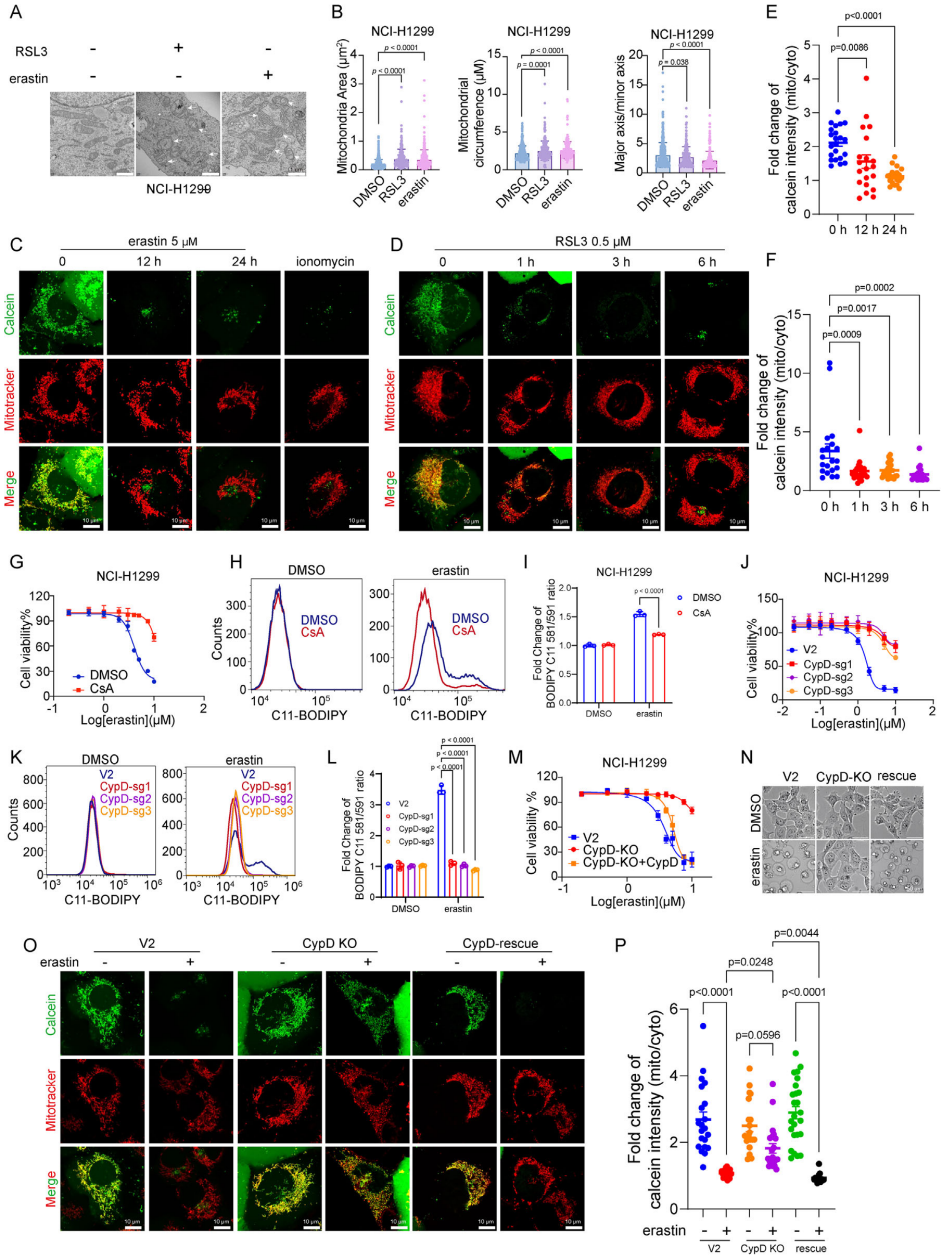
CypD is essential for the mitochondrial swelling during ferroptosis activation. (A) Transmission electron microscopy analysis of mitochondria in NCI‐H1299 treated with RSL3 or erastin. Arrows indicated the mitochondria. Scale bar was 1 µm. (B) Quantitative analysis of mitochondria area (left), mitochondrial circumference (middle), and the ratio between major axis and minor axis (right) in (A), ROI = 60, 23, 31, respectively. (C) Calcein‐CoCl2/MitoTracker fluorescence in NCI‐H1299 cells treated with erastin for the indicated period. Cells treated with ionomycin (0.5 h) were used as a positive control. The scale bar represents 10 µm. (D) Calcein‐CoCl2/MitoTracker fluorescence in NCI‐H1299 cells treated with RSL3 for the indicated period. The scale bar represents 10 µm. (E) Quantitative analysis of the fold change of Calcein fluorescence intensity within mitochondria/cytosol in each cell in (A), cell counts = 22, 20, 20, respectively. (F) Quantitative analysis of the fold change of Calcein fluorescence intensity within mitochondria/cytosol in each cell in (C), cell counts = 20, 22, 21, 20, respectively. (G) NCI‐H1299 cells were treated with increased doses of erastin as indicated in the presence of Cyclosporine A (5 µm) for 24 h, then the cell survival rate was measured by Cell Titer Glo Viability Assay. (H) NCI‐H1299 cells were subjected to 5 µm erastin for 18 h. Lipid peroxidation was measured with C11‐BODIPY staining by flow cytometry. (I) Quantitative analysis of the fold change of lipid oxidation ratio in (H). (J) Cell death of NCI‐H1299 WT and CypD KO cells subjected to the indicated doses of erastin for 24 h. (K) NCI‐H1299 WT and CypD KO cells were subjected to 5 µm erastin for 18 h. Lipid peroxidation was measured with C11‐BODIPY staining by flow cytometry. (L) Quantitative analysis of the fold change of lipid oxidation ratio in (K). (M) Cell death of NCI‐H1299 WT, CypD KO, and CypD KO supplemented with CypD cells subjected to indicated doses of erastin for 24 h. (N) The morphology of NCI‐H1299 control, CypD knockout, and CypD KO supplemented with CypD cells treated with erastin (5 µm) for 24 h. Scale bar was 50 µm. (O) Calcein‐CoCl2/MitoTracker fluorescence in NCI‐H1299 control, CypD knockout, and CypD KO supplemented with CypD cells treated with DMSO or erastin (5 µm) for 24 h. The scale bar represents 10 µm. (P) Quantitative analysis of the fold change of Calcein fluorescence intensity within mitochondria/cytosol in each cell in (O), cell counts = 21, 19, 19, 21, 25, 17, respectively. The statistical significance in (B, E, F, and P) between different groups was analyzed by One‐way ANOVA (Prism; GraphPad). Mean fluorescence intensities were analyzed with ImageJ. Two‐Way ANOVA was used for (I, L).

To investigate the function of mitochondria in the ferroptosis process, we first checked the morphological change of mitochondria during erastin/RSL3‐induced ferroptosis. Transmission Electron Microscopy (TEM) showed that mitochondria exhibited remarkable swelling in cells subjected to erastin or RSL3 treatment (Figure 1A, B), suggesting the mitochondrial permeability change in ferroptosis. Therefore, we monitored the mitochondrial permeability change by calcein–cobalt quenching assay. Under basal conditions, mitochondrial calcein fluorescence colocalized with MitoTracker, indicating normal calcium retention and a closed mitochondrial permeability state (Figure 1C–F). Upon treatment with ferroptosis inducers such as erastin or RSL3, the mitochondrial calcein signal was markedly quenched by cobalt, reflecting decreased calcium retention in mitochondria. These results suggest that ferroptosis is accompanied by upregulation of mitochondrial permeability.

As mPTP plays a pivotal role in mitochondrial permeability, we initially explored the effect of the mPTP inhibitor, Cyclosporin A (CsA), on ferroptosis. We found that erastin‐induced ferroptosis in NCI‐H1299 cells was substantially inhibited by CsA (Figure 1G). The same effect was observed in multiple cell lines, including MEF, HT1080, and others (Figure S1A–G). Lipid peroxidation accumulation was also remarkably decreased by CsA treatment (Figure 1H, I).

CsA binds with Cyclophilin D (CypD, encoded by PPIF) to impede the opening of the mPTP [43]. To confirm whether the effect of CsA on ferroptosis was derived from the modulation of mPTP, we employed the CRISPR‐Cas9 system to knock out CypD. As anticipated, the absence of CypD conferred cellular resistance to erastin‐induced ferroptosis (Figure 1J). Concurrently, it was observed that cells lacking CypD maintained cellular integrity when subjected to erastin‐induced ferroptosis (Figure 1G). Similar results were obtained when ferroptosis was induced by Class I ferroptosis inducers directly inactivating GPX4, including RSL3, ML210, and FINO2 (Figure S1H–J) [44]. Moreover, when ferroptosis was triggered by introducing an excess of polyunsaturated fatty acids (PUFAs) like arachidonic acid or adrenic acid into cell cultures, CypD deficiency also conferred resistance to ferroptosis (Figure S1K, L). Consistent with these observations, the accumulation of lipid peroxidation was also reduced (Figure 1K, L).

To further validate the role of CypD in ferroptosis, FLAG‐tagged CypD was reintroduced into CypD knockout NCI‐H1299 cells (Figure S1M), followed by subjecting the cells to ferroptosis inducers. The outcomes demonstrated that the expression of CypD reinstated their susceptibility to ferroptosis induction (Figure 1M, N, Figure S1N). Moreover, the accumulation of lipid peroxidation triggered by erastin was also reversed upon the re‐expression of CypD (Figure S1O). Furthermore, we observed that the decreased calcium retention and mitochondrial swelling responses to erastin or RSL3 treatment were also shielded by CyPD deficiency, and this effect could be rescued by CypD re‐expression (Figure 1O, P, Figure S1P, Q). Collectively, these data suggest that ferroptosis execution is mediated by CyPD, possibly through a mechanism related to mPTP.

### 2.2 mPTP Components are Involved in Ferroptosis

**FIGURE 2:**
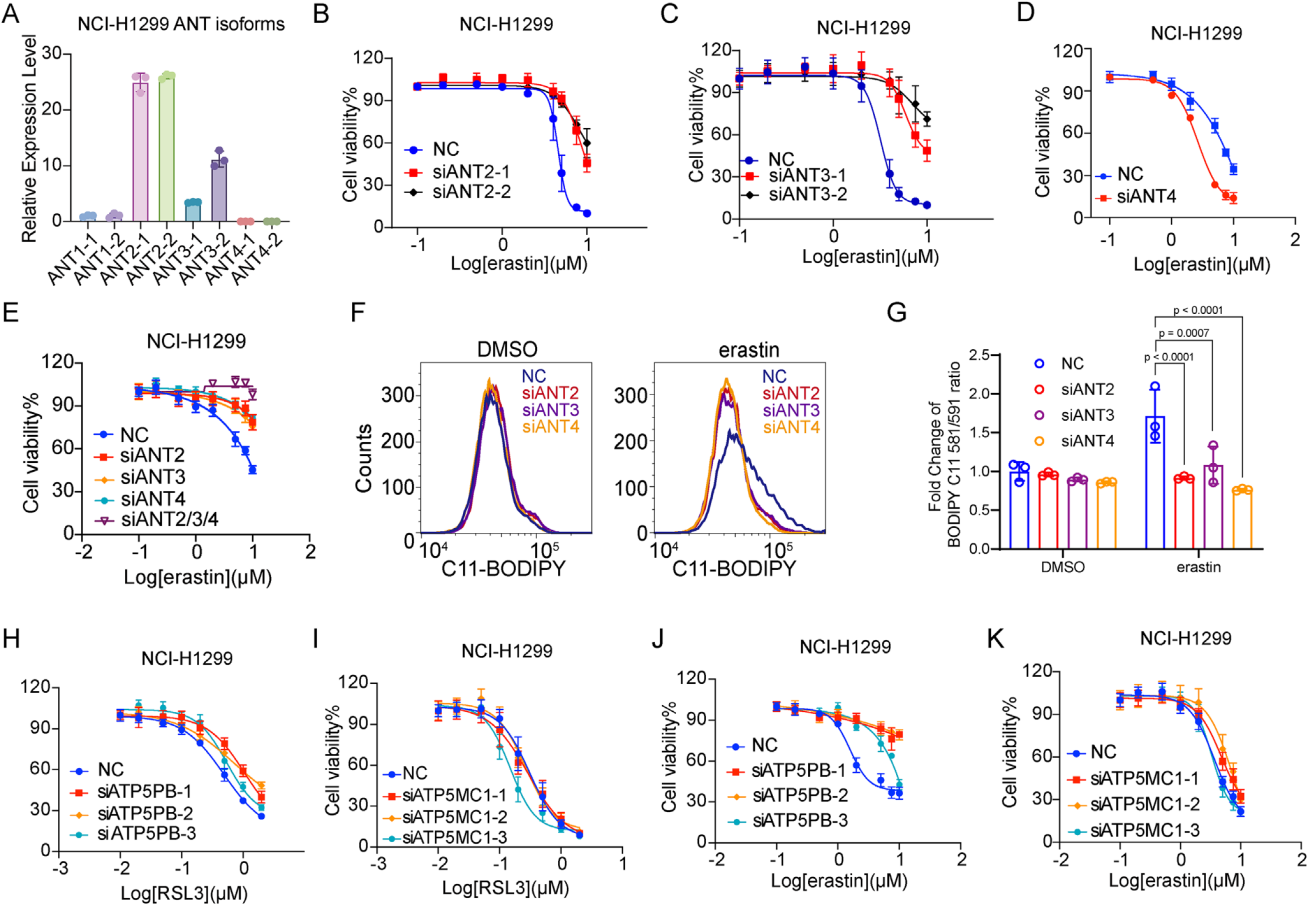
Mitochondrial permeability transition pore contributes to ferroptosis. (A) qPCR analysis of the expression level of ANT isoforms, ANT1, ANT2, ANT3, and ANT4 in NCI‐H1299 cells. The expression of each gene was analyzed using two different pairs of primers. (B–D). ANT2 (B), ANT3 (C), or ANT4 (D) was respectively knocked down by specific siRNA in NCI‐H1299 cells, followed by indicated doses of erastin for 24 h to detect cell viability. (E) ANT2, ANT3, and ANT4 were knocked down simultaneously by specific siRNA in NCI‐H1299 cells, followed by indicated doses of erastin for 24 h to detect cell viability. (F) NCI‐H1299 cells were transfected with siRNA specifically against ANT2, ANT3, and ANT4 for 48 h, then subjected to 5 µm erastin for 18 h. Lipid peroxidation was measured with C11‐BODIPY staining by flow cytometry. (G) Quantitative analysis of the fold change of lipid oxidation ratio in (F). (H, I) ATP5PB (H) and ATP5MC1 (I) were knocked down by specific siRNA in NCI‐H1299 cells, followed by indicated doses of RSL3 for 8 h to detect cell viability. (J, K) ATP5PB (J) and ATP5MC1 (K) were knocked down by specific siRNA in NCI‐H1299 cells, followed by indicated doses of erastin for 24 h to detect cell viability. Two‐Way ANOVA was used for (G).

In the past few decades, the theoretical framework of mPTP has been continuously evolving, with various proteins being added to or removed from this model. The latest model includes mitochondrial F1/F0 ATP synthase subunit OSCP (Oligomycin sensitivity‐conferring protein, hereafter referred to as ‘ATP synthase’), CypD, and ANT (Adenine Nucleotide Translocator) [45]. Next, we tried to establish the role of mPTP in ferroptosis through intervening in the complex by chemical and genetic suppression of its components. It is reported that adenine nucleotide translocase (ANT), together with CypD, facilitates the opening of mPTP [46]. We first detected expression of different ANT isoforms in NCI‐H1299 cells. Quantitative PCR results suggested that ANT2 and ANT3, but not ANT1, were highly expressed in NCI‐H1299 cells (Figure 2A). Next, we tried to figure out whether ANT isoforms contribute to ferroptosis. While ANT1 knockdown did not manifest a protective effect in response to ferroptosis induction, we observed that siRNA‐mediated ANT2/3/4 knockdown resulted in increased resistance to erastin or RSL3‐induced ferroptosis (Figure 2B, D and Figure S2A–I). Triple knockdown of ANT2, ANT3, and ANT4 further prevented ferroptosis in NCI‐H1299 cells with erastin or RSL3 treatment (Figure 2E and Figure S2J). Meanwhile, erastin‐induced lipid peroxidation was also dampened in ANT2/3/4 knockdown cells (Figure 2F, G).

ATP synthase subunits, particularly the B subunit (ATP5PB, Ca^2^^+^ binding domain) and C subunit (ATP5MC1, pore‐forming protein), have been reported to participate in mPTP formation [19, 47]. Three independent siRNAs were used to knock down ATP5PB and ATP5MC1, and the knockdown efficiency is shown in Figure S2K, L. Upon RSL3 treatment, knockdown of either ATP5PB or ATP5MC1 did not alter cellular sensitivity to ferroptosis (Figure 2H, I). Under erastin treatment, only ATP5PB knockdown showed a protective effect against ferroptosis (Figure 2J, K). These results suggest that ferroptosis‐associated mPTP opening may preferentially involve ANT‐dependent rather than ATP‐synthase‐dependent pathways.

The mitochondrial matrix ATPase associated with diverse cellular activities (m‐AAA) protease spastic paraplegia 7 (SPG7) has been recently implicated as a positive regulator of the mPTP [48]. The interaction between AFG3L2 and SPG7 also contributes to mPTP opening [49]. To test the role of AFG3L2 and SPG7 in ferroptosis, siRNA oligos targeting SPG7 and AFG3L2 were used to knock down these protein respectively (Figure S2M, P). Knockdown of SPG7 significantly blocked ferroptosis and lipid peroxidation induced by erastin/RSL3 (Figure S2N, O, R, S). Consistent results were observed when AFG3L2 was knocked down (Figure S2P–S). These data together suggest that multiple components and regulators of mPTP are critical for ferroptosis execution.

### 2.3 mPTP Does Not Directly Interfere with Iron Metabolism or Anti‐Oxidant Production

Iron metabolism and lipid synthesis are pivotal events in the occurrence of ferroptosis. The acquisition of iron through transferrin receptor protein 1 (TFR1, also known as TFRC) or the breakdown of ferritin for iron reservoirs leads to an increase in the labile iron pool, making cells vulnerable to ferroptosis due to the generation of lipid hydroperoxides [50]. Cell susceptibility to PUFA peroxidation and subsequent cellular damage in ferroptosis is facilitated by the incorporation of PUFA into the membrane by essential enzymes such as ACSL4 and lyso‐phosphatidylcholine acyltransferase 3 (LPCAT3) [51]. In order to explore the possible influence of CypD on these vital mechanisms, we first examined the levels of important proteins related to iron and lipid metabolism as well as lipid peroxidation. This analysis included assessing the expression of LPCAT3, ACSL4, FSP1, GPX4, DHODH, and TFRC. Interestingly, we did not detect any changes in the expression of these proteins following the removal of CypD (Figure S3A). Meanwhile, CsA exhibited a similar protective effect in ACSL4 knockout (ACSL4 KO) cells, excluding the possible inhibitory effect of mPTP on PUFA synthesis (Figure S3B). Subsequently, we investigated whether the intracellular labile iron pool decreased when CypD was absent. Flow cytometry analysis indicated that the level of labile Fe^2+^ remained unaltered in the absence of CypD, contrasting with the notable decrease observed with deferoxamine (DFO) treatment, which substantially decreased the levels of ferrous iron (Figure S3C). Consistently, we did not observe any regulatory effect of CsA or ANT knockdown on iron levels within living cells (Figure S3D–F).

An anti‐oxidative compound leads to ferroptosis inhibition directly. CsA did not demonstrate any antioxidant activity in the DPPH assay either (Figure S3G). Glutathione (GSH), the intracellular antioxidant, decreases in ferroptosis and thus promotes lipid peroxidation [52]. Our results indicated that CypD had no impact on GSH levels, which decrease following erastin or BSO treatment (Figure S3H). Collectively, our results indicate that mPTP does not directly interfere with iron metabolism or antioxidant production.

### 2.4 Oxidized mtDNA Release Through mPTP Contributes to Ferroptosis

mPTP opening facilitates the free passage of small molecules like calcium, ROS, and fragmented DNAs into the cytosol [19, 53, 54]. Since mPTP has no effect on iron metabolism or antioxidant production, we aimed to explore whether the opening of mPTP releases specific signaling molecules, such as peptides or oligos that promote the process of ferroptosis.

**FIGURE 3:**
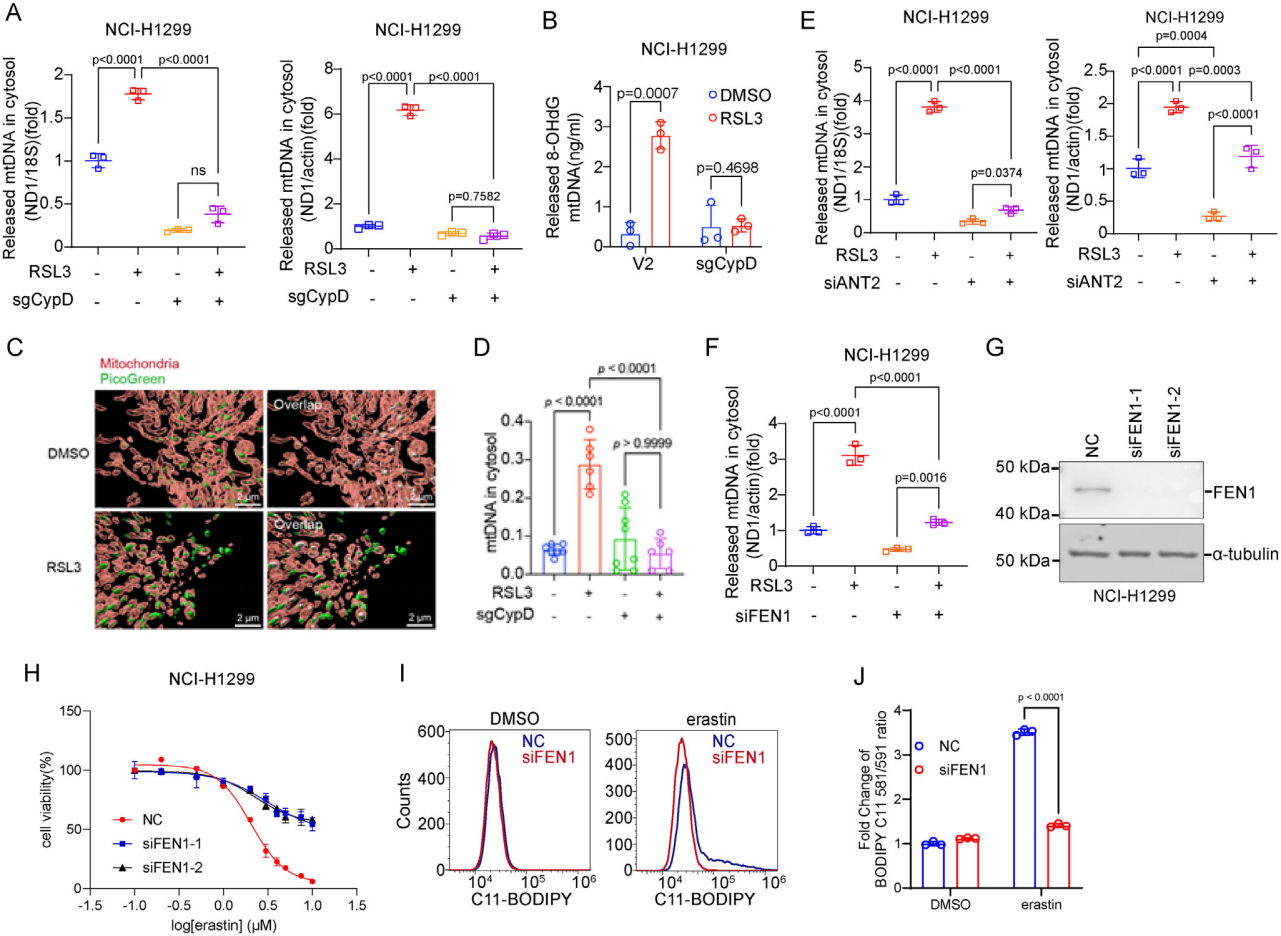
MtDNA release mediated by mPTP promotes ferroptosis. (A) Relative cytosolic mtDNA amounts in RSL3‐treated NCI‐H1299 WT and CypD‐KO cells. The relative ratios of ND1 mtDNA, 18S nuclear DNA (left panel), and actin (right panel) are shown. (B) Relative amounts of Ox‐mtDNA in cytosols of RSL3‐primed WT or CypD‐KO NCI‐H1299 cells. (C) Representative 3D reconstruction images showing mitochondria (red, MitoTracker) and mitochondrial DNA (green, PicoGreen) in cells treated with DMSO or RSL3. Super‐resolution images were acquired using structured illumination microscopy (SIM). Mitochondria structures were labeled in red, and mtDNA signals were labeled in green (left panels). The mtDNA signals that were in contact with the mitochondrial surface were labeled in light pink, while those not in contact with the mitochondrial surface were in green (right panels, shown in the overlap images). Scale bar was 2 µm. (D) Quantification of cytosolic mtDNA (mtDNA in cytosol) in indicated groups. (E, F) ANT2 (E) and FEN1 (F) were knocked down by specific siRNA in NCI‐H1299 cells, followed by RSL3 treatment for 6 h to detect relative cytosolic mtDNA. (G) Western Blot analysis of FEN1 knockdown cells. (H) FEN1 was knocked down by specific siRNA in NCI‐H1299 cells, followed by indicated doses of erastin for 24 h to detect cell death. (I) NCI‐H1299 cells were transfected with siRNA specifically against FEN1 for 48 h, then subjected to 5 µm erastin for 18 h. Lipid peroxidation was measured with C11‐BODIPY staining by flow cytometry. (J) Quantitative analysis of the fold change of lipid oxidation ratio in (I). The statistical significance between different groups (A, D, E, and F) was analyzed by One‐way ANOVA (Prism; GraphPad). Two‐Way ANOVA was used for (B and J).

It was reported that mitochondrial DNA (mtDNA) is released into the cytosol in response to oxidative stress H_2_O_2_ stimulation, triggering an inflammatory response [54]. mtDNA fragments shorter than 700 base pairs can be released into the cytosol via mPTP. We examined whether mtDNA fragments are released during ferroptosis. We observed that NCI‐H1299 cells treated with RSL3, erastin, or ML210 exhibited elevated levels of cytoplasmic mitochondrial DNA (Figure 3A, Figure S4A, B). Meanwhile, CypD deficiency prevented the release of mtDNA, suggesting that mPTP mediated the release of mtDNA during ferroptosis (Figure 3A, Figure S4A, B).

Additionally, reactive oxygen species (ROS) could trigger oxidative base lesions in mtDNA and are widely recognized to induce the accumulation of mtDNA mutations during the aging process and in various disease conditions [55]. In our previous research, we observed that ferroptosis is associated with an elevation of mitochondrial reactive oxygen species (ROS) production and heightened lipid peroxidation within mitochondria [29]. This led us to contemplate the concurrent occurrence of oxidative damage to mitochondrial DNA. As expected, NCI‐H1299 cells treated with RSL3 exhibited an elevated level of oxidized mtDNA in the cytosol, while there was a significant decrease in the release of oxidized mtDNA in CypD knockout cells, suggesting that oxidized mtDNA was discharged through mPTP in the process of ferroptosis (Figure 3B). Next, we employed the DNA‐binding dye PicoGreen to visualize fragmented DNA near mitochondria using super‐resolution microscopy. 3D surface reconstructions were generated with Imaris software, revealing that, upon RSL3 treatment, mtDNA signals were redistributed from mitochondria into the cytoplasm (Figure 3C). Cytosol mtDNA signals were diminished in CypD knockout cells (Figure 3D). In order to provide further validation that mtDNA release is facilitated via mPTP, we investigated these occurrences within the context of ANT2/SPG7 knockdown. Crucially, the suppression of mitochondrial DNA release induced by RSL3 in NCI‐H1299 cells was also observed upon the knockdown of ANT2 and SPG7 (Figure 3E, Figure S4C). Taken together, these results suggest that mPTP mediates the release of mitochondrial DNA during the process of ferroptosis.

To confirm the contribution of the mtDNA release to ferroptosis, we employed approaches to manipulate mtDNA release and tested whether the cell sensitivity to ferroptosis was changed. Flap‐structure‐specific endonuclease 1 (FEN1) participates in repairing mitochondrial oxidative DNA damage and maintaining mitochondrial DNA integrity [54]. FEN1 is required for efficient oxidized bases excision from the DNA double strand [56, 57]. Loss of FEN1 leads to fewer free oxidized DNA fragments under oxidative stress conditions. We observed that knockdown of FEN1 reduced mitochondrial DNA release (Figure 3F, G), blocked erastin‐induced ferroptosis (Figure 3H), and accumulation of lipid ROS (Figure 3I, J). Consistently, the FEN1 inhibitor FEN‐IN‐4 also showed dose‐dependent inhibition of ferroptosis induction while reducing the level of lipid ROS (Figure S4D–F). These results demonstrate that the oxidized mtDNA (ox‐mtDNA) released through mPTP contributes to ferroptosis.

### 2.5 Accumulation of Cytoplasmic Ox‐mtDNA Aggravates Ferroptosis

**FIGURE 4:**
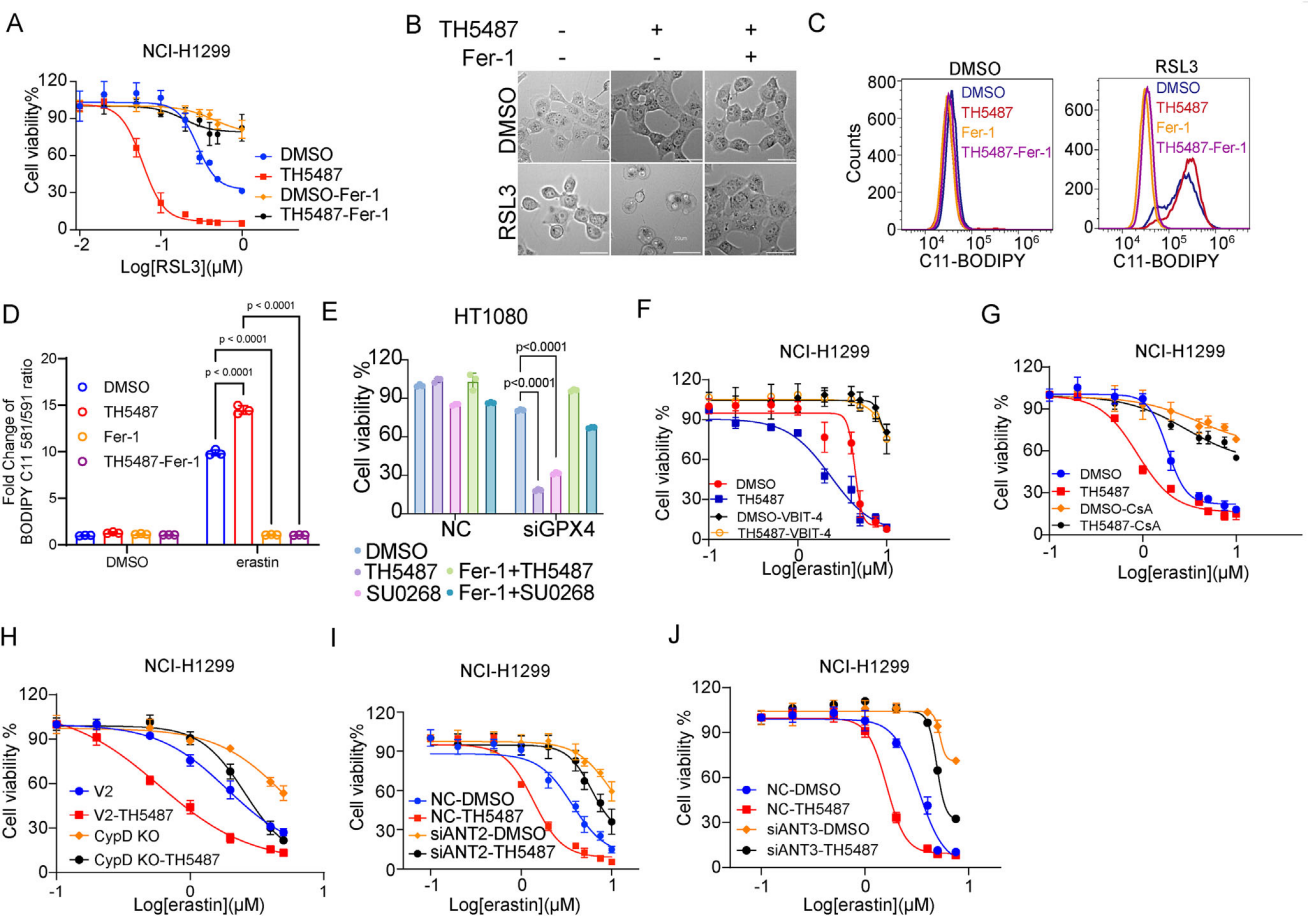
OGG1 inhibition promotes ferroptosis via an mPTP‐dependent mechanism. (A) NCI‐H1299 cells were treated with increased doses of RSL3 as indicated in the presence of TH5487 (5 µm) and Fer‐1 (2 µm) for 8 h; then cell survival rate was measured by Cell Titer Glo Viability Assay. (B) The morphology of NCI‐H1299 cells treated with RSL3 (0.5 µm) for 4 h following TH5487 (5 µm) and Fer‐1 (2 µm) pre‐treatment. Scale bar was 50 µm. (C) NCI‐H1299 were treated with 5 µm erastin as indicated in the presence of TH5487 (5 µm) and Ferrostatin (Fer‐1, 2 µm) for 18 h, and the lipid peroxidation level was determined using C11‐BODIPY staining by flow cytometry. (D) Quantitative analysis of the fold change of lipid oxidation ratio in (C). (E) GPX4 siRNA were transfected to HT‐1080 cells, after 36 h, TH5487, SU0268 and Fer‐1 were added as indicated, then cells viability was detected using Cell Titer Glo. (F) NCI‐H1299 were treated with increased doses of erastin as indicated in the presence of TH5487 (5 µm), with or without VBIT‐4 (5 µm) pretreatment for 8 h, then cell survival rate was measured by Cell Titer Glo Viability Assay. (G) NCI‐H1299 cells were treated with increased doses of erastin as indicated in the presence of TH5487 (5 µm), with or without CsA (5 µm) pretreatment for 24 h, and then the cell survival rate was measured by Cell Titer Glo Viability Assay. (H) CypD were knocked out in NCI‐H1299 cells, then the cells were treated with increased doses of erastin with or without TH5487 (5 µm) pretreatment. Cell death was measured by Cell Titer Glo Viability Assay. (I, J) ANT2 (I) and ANT3(J) were knocked down by specific siRNA in NCI‐H1299 cells, then the cells were treated with increased doses of erastin with or without TH5487 (5 µm) pretreatment. Cell death was measured by Cell Titer Glo Viability Assay. The statistical significance between different groups (D and E) was analyzed by Two‐Way ANOVA (Prism; GraphPad).

8‐oxoguanine DNA glycosylase (OGG1) functions as the primary glycosylase catalyzing the excision of 8‐oxoG from double‐stranded DNA to initiate base excision repair (BER) and reduce the buildup of oxidized mtDNA [58]. Considering the impact of mtDNA release on ferroptosis, we aimed to investigate whether OGG1 inhibition exaggerated the accumulation of oxidized mtDNA released through the mPTP and subsequent ferroptosis. We employed TH5487, a potent small‐molecule active‐site inhibitor of OGG1, to disturb mtDNA repair [58]. The addition of TH5487 significantly exacerbated cell death in NCI‐H1299 cells, which could be completely reversed by Fer‐1 and DFO (Figure 4A, B, Figure S5A, B). Consistently, treatment with TH5487 remarkably increased the accumulation of lipid ROS (Figure 4C, D). Similar to RSL3‐induced ferroptosis, ferroptosis triggered by GPX4 knockdown was also potentiated by TH5487 treatment (Figure 4E). Furthermore, SU0268, identified as a selective inhibitor of OGG1 [59], also intensified RSL3‐induced ferroptosis in NCI‐H1299 cells, which could be fully reversed by Fer‐1 (Figure S5C–E). Similar results were obtained in MEF cells (Figure S5F, G).

Next, we investigated whether the exacerbation of ferroptosis due to OGG1 inhibition is contingent on mtDNA release through the mPTP. On the outer membrane of mitochondria, VDAC1 oligomerizes to create pores that facilitate the release of mtDNA [54]. We utilized a small molecule that targets VDAC1 oligomerization to investigate whether the ferroptosis induced by OGG1 inhibition was sustained. The administration of TH5487 significantly augmented ferroptosis, a process that could be entirely reversed by the VDAC1 oligomerization inhibitor VBIT‐4 (Figure 4F, Figure S5H).

Next, we validated the role of the mPTP in this event. Both chemical and genetic inhibition of CypD effectively countered the exacerbating effect of OGG1 inhibition on ferroptosis (Figure 4G, H, Figure S5I—K). Regarding other components or regulators of the mPTP, ANT2 (Figure 4I, Figure S5L), ANT3 (Figure 4J, Figure S5M), SPG7 (Figure S5N, O), and AFG3L2 (Figure S5P) were knockdown respectively, the magnifying effect of OGG1 inhibition on ferroptosis was effectively counteracted. Taken together, OGG1 inhibition results in the accumulation of oxidized mtDNA released through mPTP, thereby amplifying ferroptosis.

### 2.6 OGG1 Inhibition Enhances Ferroptosis via cGAS‐Dependent Signaling Pathway

**FIGURE 5:**
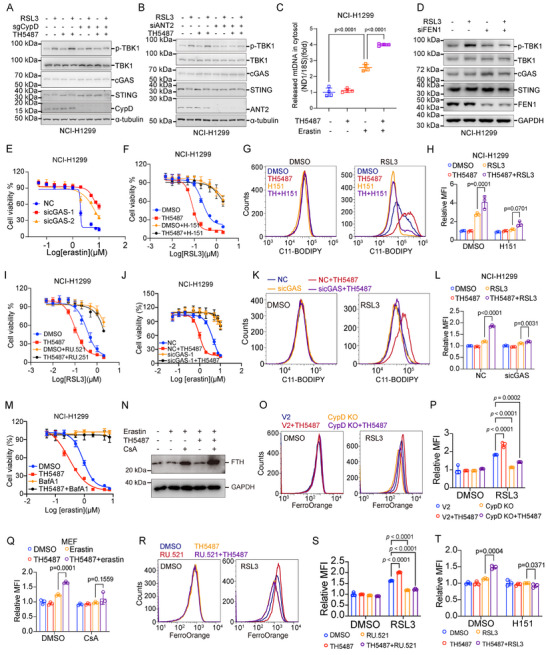
OGG1 inhibition exacerbates ferroptosis through the cGAS‐STING pathway. (A, B) CypD (A) was knocked out, and ANT2 (B) was knocked down. Immunoblot analysis of TBK1 Ser172 phosphorylation and TBK1, STING, and cGAS of RSL3‐primed NCI‐H1299 that were pretreated ±TH5487 (5 µm). (C) Relative cytosolic mtDNA amounts in erastin‐treated (5 µm, 18 h) NCI‐H1299 cells in the presence/absence of TH5487 treatment (5 µm). The relative ratios of ND1 mtDNA and 18S nuclear DNA are shown. (D) FEN1 was knocked down by specific siRNA in NCI‐H1299 cells for 48 h, then the cells were treated with RSL3 for 6 h. Western blot analysis of TBK1 Ser172 phosphorylation and TBK1, STING, and cGAS in cells with the indicated treatment. (E) cGAS was knocked down by specific siRNA in NCI‐H1299 cells, followed by indicated doses of erastin for 24 h to detect cell viability. (F) NCI‐H1299 cells were treated with increased doses of RSL3 as indicated in the presence of H‐151 (1 µm) for 6 h, then the cell survival rate was measured by Cell Titer Glo Viability Assay. (G) NCI‐H1299 cells were treated with RSL3 as indicated in the presence of TH5487 (5 µm) and H‐151 (1 µm) for 6 h, and the lipid peroxidation level was determined using C11‐BODIPY staining by flow cytometry. (H) Quantitative analysis of the fold change of lipid oxidation ratio in (G). (I) NCI‐H1299 cells were treated with increased doses of RSL3 as indicated in the presence of RU.521 (10 µm) for 8 h, then the cell survival rate was measured by Cell Titer Glo Viability Assay. (J) cGAS was knocked down by specific siRNA in NCI‐H1299 cells, then the cells were treated with increased doses of RSL3 with or without TH5487 (5 µm) pretreatment, and then the cell survival rate was measured by Cell Titer Glo Viability Assay. (K) cGAS was knocked down by specific siRNA in NCI‐H1299 cells, then the cells were treated with RSL3 with or without TH5487 (5 µm) pretreatment, and the lipid peroxidation level was determined using C11‐BODIPY staining by flow cytometry. (L) Quantitative analysis of the fold change of lipid oxidation ratio in (K). (M) NCI‐H1299 cells were treated with increased doses of erastin as indicated in the presence of TH5487 (5 µm) and Bafilomycin A1 (BafA1, 100 nm) for 24 h, then the cell survival rate was measured by Cell Titer Glo Viability Assay. (N) Western Blot analysis of FTH1 in MEF cells treated with erastin (5 µm, 6 h) in the presence of CsA (5 µm) or TH5487 (5 µm). (O) NCI‐H1299 WT, CypD KO cells were treated as indicated, then the labile iron level was detected by FACS using FerroOrange. (P) Quantitative analysis of mean fluorescence intensity for (O). (Q) NCI‐H1299 cells were treated as indicated, then the labile iron level was detected by FACS using FerroOrange. Quantitative analysis of mean fluorescence intensity was shown. (R) NCI‐H1299 cells were treated with increased doses of RSL3 as indicated in the presence of RU.521 (10 µm) for 6 h, then the labile iron level was detected by FACS using FerroOrange. (S) Quantitative analysis of mean fluorescence intensity for (R). (T) NCI‐H1299 cells were treated with RSL3 as indicated in the presence of TH5487 (5 µm) and H‐151 (1 µm) for 4 h, then the labile iron level was detected by FACS using FerroOrange. Quantitative analysis of mean fluorescence intensity was shown. The statistical significance between different groups (C) was analyzed by One‐Way ANOVA. Two‐Way ANOVA analysis was used in other statistics (Prism; GraphPad).

The release of mitochondrial DNA into the cytoplasm is recognized by cGAS, leading to the phosphorylation of STING and activation of downstream immune responses [60]. To determine whether mPTP is involved in the activation of the cGAS‐STING pathway upon ferroptotic induction, the phosphorylation of TBK1 was analyzed in different conditions. We observed that RSL3 treatment enhanced the phosphorylation of TBK1, and the phosphorylation can be inhibited by Fer‐1 treatment, suggesting that the cGAS‐STING signaling pathway was activated upon ferroptosis (Figure 5A, B, Figure S6A, B). Moreover, OGG1 inhibition with TH5487 or SU0268 enhanced the phosphorylation of TBK1 to a greater extent (Figure 5A, B, Figure S6A, B). Depletion of CypD or knockdown of either ANT2 or SPG7attenuated TBK1 activation (Figure 5A, B, Figure S6A, B). Furthermore, we observed more released mtDNA in the cytosol with the combined treatment of TH5487 plus erastin (Figure 5C). Meanwhile, ferroptosis‐induced activation of cGAS‐STING was markedly attenuated in FEN1‐knocked‐down cells, suggesting that smaller mtDNA fragments are more efficiently released into the cytoplasm to activate cGAS‐STING (Figure 5D). These results suggest that the activation of the cGAS‐STING pathway during ferroptosis depends on fragmented mtDNA release through mPTP opening.

Along with cGAS‐STING pathway activation, the RNA‐seq results indicated that several immune pathways were activated under RSL3 treatment in WT cells but not in CypD knockout cells (Figure S6C, D), indicating that inflamation response were elicited upon ferroptosis in an mPTP‐dependent manner.

The cGAS‐STING pathway was demonstrated to be involved in ferroptosis [61]. We therefore confirmed the necessity of the cGAS‐sting pathway in ferroptosis through small molecule inhibitors targeting cGAS or STING, respectively. STING inhibitor H‐151 (Figure S6E) and cGAS inhibitor RU.521 (Figure S6F) remarkably inhibited RSL3 or erastin‐induced ferroptosis. Similar results were obtained when cGAS was knocked down with specific siRNA (Figure 5E, Figure S6G). Moreover, TH5487 caused exacerbation in either RSL3 or erastin‐induced ferroptosis and lipid peroxidation, which were attenuated by the content of cGAS or STING inhibition (Figure 5F, H, Figure S6H, I). Similar results were obtained when cGAS was knocked down with specific siRNA (Figure 5J–L, Figure S6J). These data suggest that OGG1 inhibition exacerbates ferroptosis via activation of the cGAS‐STING pathway.

### 2.7 mtDNA Damage Accelerates Ferroptosis by Labile Iron Release via Ferrotinophagy

It was reported that cGAS‐STING activation potentiates ferroptosis via ferrotinophagy, which leads to the release of free iron and thus an increase in the labile iron pool (LIP) [62, 63, 64, 65, 66]. Therefore, we wondered whether TH5487‐induced exacerbation of ferroptosis was mediated by ferrotinophagy. To address this issue, autophagosomal degradation inhibitor Bafilomycin A1 (Baf A1) was used to block ferrotinophagy. We observed that TH5487‐induced exacerbation of ferroptosis was strongly inhibited in cells subjected to BafA1 treatment (Figure 5M). Consistent results were obtained in HT1080 cells (Figure S6K, L) and MEF cells (Figure S6M, N). In addition, we analyzed the expression of ferrotinophagy marker protein FTH1 by western blot under ferroptosis induction with CsA and TH5487 treatments. CsA clearly suppressed the degradation of FTH1, indicating a reduction of ferritinophagy upon mPTP inhibition (Figure 5N). The STING inhibitor H‐151 also dampened FTH1 degradation, implying that cGAS–STING signaling contributes to the regulation of ferritinophagy (Figure S6O). Meanwhile, we detected LIP in TH5487‐treated cells. LIP was further accumulated in cells upon TH5487 treatment, and this was fully blocked by CypD knockout (Figure 5O, P), CsA (Figure 5Q), cGAS (Figure 5R, S), or STING inhibition (Figure 5T, Figure S6P) treatment. These results suggest that TH5487‐induced exacerbation of ferroptosis is dependent on ferrotinophagy downstream of cGAS‐STING signal.

### 2.8 OGG1 Inhibitor Synergizes with Ferroptosis Inducer in Cancer Therapy

**FIGURE 6:**
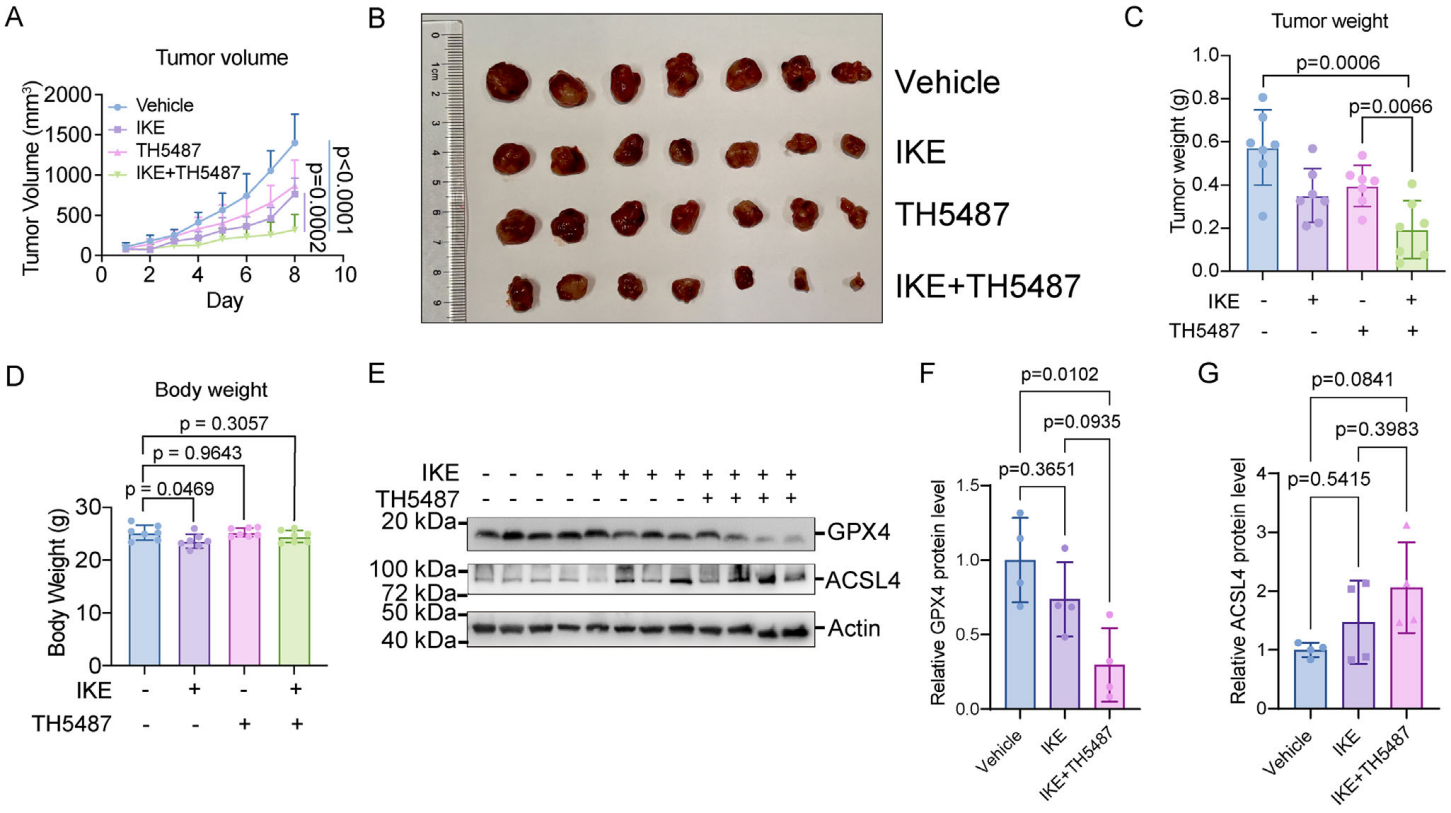
OGG1 inhibition exhibited synergistic anti‐tumor effects with ferroptosis inducer IKE in vivo. (A) Growth curves of xenograft tumors derived from NCI‐H1299 cells. Tumor‐bearing mice were randomly divided into 4 groups and treated intraperitoneally with the indicated compounds for 9 days. (B, C) Images (B) and tumor weight (C) are shown at day 9. The tumor sizes were measured every day. (D) Body weight of mice at day 9. (E) Western blot analysis of GPX4 and ACSL4 in xenograft tumors with indicated treatment. (F, G) Statistical analysis of the protein levels from specific blots in (E). The statistical significance between different groups was analyzed by One‐way ANOVA (Prism; GraphPad).

Considering the significant exacerbating effect of OGG1 inhibitor on ferroptosis, we hypothesized that TH5487 might synergize with ferroptosis inducer in cancer therapy. Imidazole ketone erastin (IKE) is a potent, metabolically stable inhibitor of system xc‐ and inducer of ferroptosis, potentially suitable for in vivo applications [67]. Therefore, we tested the effect of TH5487 on sensitizing the IKE‐mediated killing effect in vivo. We generated a xenograft model in BALB/c‐nu/nu mice with subcutaneous inoculation of NCI‐H1299 cells, followed by IKE alone, TH‐5487 alone, or IKE plus TH5487 treatment. While IKE or TH5487 alone restrained tumor growth to a small extent, IKE plus TH5487 further decreased tumor growth rate and tumor size (Figure 6A–C) without obvious change in body weight (Figure 6D). Moreover, we performed western blot analysis of xenograft tumor lysates to evaluate ferroptosis‐related markers. The results showed a marked decrease in Glutathione Peroxidase 4 (GPX4) and a concomitant increase in Acyl‐Coenzyme A Synthetase Long‐chain 4 (ACSL4) expression in the IKE+TH5487‐treated tumors compared with the control and IKE group, indicating the exacerbation of ferroptosis (Figure 6E–G). Our data indicates the synergistic effect of the OGG1 inhibitor on ferroptosis inducer and suggests potential cancer therapy regarding ferroptosis and drug combination.

## 3 Discussion

**FIGURE 7:**
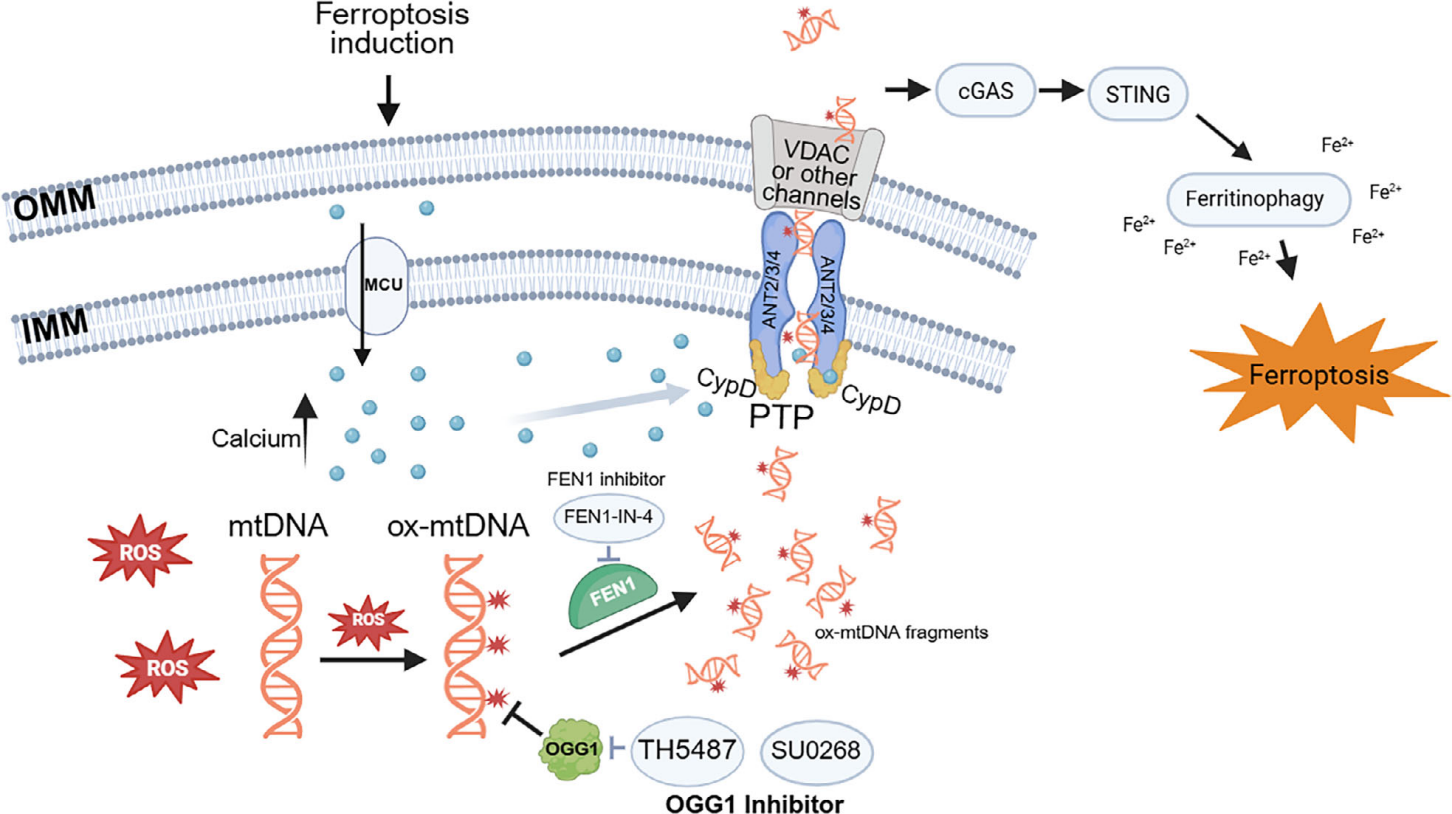
Schematic diagram of mPTP‐involved ferroptosis.

Ferroptosis, a form of regulated cell death driven by lipid peroxidation, involves mitochondrial ROS production and membrane potential changes. However, the signaling mechanisms linking mitochondria to cytoplasmic cell death and immune responses remain unclear. Our study reveals that mitochondrial permeability transition pore (mPTP) opening is essential for ferroptosis, facilitating the release of oxidized mitochondrial DNA (ox‐mtDNA). These mtDNAs act as damage‐associated molecular patterns (DAMPs), activating the inflammatory response and cGAS‐STING pathway to amplify ferroptosis. Furthermore, mtDNA damage enhances cellular ferroptosis sensitivity, highlighting the drug combination of ferroptosis inducer and mtDNA repair inhibitor as a potential cancer therapy (Figure 7).

The mitochondrial permeability transition pore (mPTP) is a pivotal regulator of cell death, playing distinct roles in apoptosis, necrosis, and other forms of regulated cell death [19, 68, 69, 70, 71, 72]. In apoptosis, transient or low‐level mPTP opening can lead to the release of pro‐apoptotic factors such as cytochrome c, initiating caspase activation and controlled cell dismantling [73]. In contrast, sustained mPTP opening disrupts mitochondrial membrane potential and promotes matrix swelling, ultimately leading to mitochondrial rupture and necrosis [19, 71, 74]. mPTP ‐driven necrosis has long been recognized as an unregulated form of cell death in the context of ischemia‐reperfusion injury (IRI), while the precise mechanisms remain incompletely understood [75, 76, 77]. Our study extends the role of mPTP beyond apoptosis and necrosis to ferroptosis. This may help to illustrate the reason why inhibition of mPTP via CsA exhibits a protective effect in IRI [43, 78]. The study also reveals a previously unappreciated link between mitochondrial dysfunction, immunogenic signaling, and ferroptosis. Multiple small molecules, including calcium, ROS, and other metabolites, have been reported to exhibit as subsequences of mPTP to play crucial roles in cell fate determination [19, 79]. Our findings suggest that mPTP serves as a central hub coordinating not only metabolic and redox stress but also the mode and immunological consequences of cell death. The dual role of mPTP in regulating both cell demise and inflammatory pathways underscores its potential as a therapeutic target in diseases characterized by apoptosis, necrosis, or ferroptosis.

When ferroptosis was originally described in 2012, it was characterized as an iron‐dependent, caspase‐independent form of regulated cell death, distinct from other forms such as apoptosis and necrosis. Key features include the accumulation of lipid peroxides, particularly phospholipids containing polyunsaturated fatty acids, and the inactivation of glutathione peroxidase 4 (GPX4). This form of cell death was considered independent of mitochondrial permeability transition pore (mPTP)‐driven necrosis, which is typically associated with ATP depletion and mitochondrial dysfunction [1, 16, 17, 18, 80]. However, recent studies have revealed that ferroptosis and mPTP‐driven necrosis may not be entirely independent. For instance, research has shown that mPTP‐dependent necrosis and ferroptosis can additively contribute to infarct size following ischemia/reperfusion injury. Inhibition of mPTP was found to reduce necrotic cell death, while ferroptosis inhibitors provided additional protection, suggesting a potential crosstalk between these pathways [81]. Furthermore, other studies have indicated that mPTP opening can lead to mitochondrial dysfunction, which may influence the cellular environment in a way that promotes ferroptosis. This suggests that under certain conditions, the activation of ferroptosis and mPTP‐driven necrosis could be interconnected, rather than entirely distinct processes [82].

Mitochondrial DNA (mtDNA) has emerged as a critical mediator of cellular stress responses and cell death pathways [83, 84, 85]. Unlike nuclear DNA, mtDNA is more susceptible to oxidative damage due to its proximity to the mitochondrial electron transport chain and lack of robust repair mechanisms [86]. Under conditions of mitochondrial dysfunction, damaged mtDNA can be released into the cytoplasm, where it acts as a potent damage‐associated molecular pattern (DAMP) to amplify death signal and activate innate immune signaling pathways [87, 88, 89, 90, 91]. For instance, mtDNA released during apoptosis can enhance caspase‐independent inflammatory responses [87, 88, 92, 93], while in necrosis, it contributes to the immunogenicity of cell death by triggering toll‐like receptor (TLR) or cGAS‐STING signaling [94, 95]. Multiple channels or pore‐forming proteins have been reported to govern the release of mtDNA, including mPTP, VDAC, BAK/BAX, and gasdermins. [54, 89, 96, 97] Our findings further demonstrate the pivotal role of mPTP ‐mediated mtDNA as mitochondrial DAMP in ferroptosis.

The cGAS‐STING pathway is a well‐characterized innate immune signaling cascade that detects cytosolic double‐stranded DNA (dsDNA), including oxidized mitochondrial DNA (mtDNA), and initiates type I interferon (IFN) production and pro‐inflammatory cytokine responses [36, 98]. Traditionally, this pathway has been viewed as a sentinel of cellular stress, linking DNA damage or mitochondrial dysfunction to immune activation. However, our findings reveal a non‐canonical role of the cGAS‐STING pathway in ferroptosis, distinct from its classical immune functions. Specifically, we observed that activation of cGAS‐STING by oxidized mtDNA during ferroptosis promotes ferroptotic cell death through iron autophagy (ferrotinophagy) [64, 66].

This unexpected link suggests that cGAS‐STING signaling may function independently of its immune‐regulatory roles to modulate intracellular iron homeostasis. By facilitating ferritin degradation and subsequent iron release, cGAS‐STING amplifies ferroptosis‐associated lipid peroxidation. These findings provide a novel perspective on cGAS‐STING as a mediator of metabolic and cell death pathways, broadening its functional repertoire beyond immunity. This non‐immune function of cGAS‐STING could have significant implications for diseases where ferroptosis and iron dysregulation play key roles, such as neurodegeneration and cancer. Targeting cGAS‐STING in these contexts might offer therapeutic opportunities distinct from traditional immune modulation strategies.

Interestingly, we also observed that mPTP positively regulates immune‐associated signaling during ferroptosis. In addition to cGAS–STING, mPTP‐mediated mitochondrial perturbation may engage multiple immune signaling axes, including JAK–STAT signaling and Th1/Th2 cell differentiation pathways, to shape the inflammatory landscape of ferroptosis (Figure S6C, D). However, as our experiments were conducted in tumor cells, we did not explore the broader implications of these immune responses in detail. This raises an important question about the role of mPTP and mtDNA in shaping immune‐related pathways during ferroptosis in immune cells or in vivo. Future studies will aim to investigate how mPTP‐mediated mtDNA release influences immune cell activation and inflammatory responses during ferroptosis. This will be particularly relevant for understanding the dual roles of ferroptosis in promoting tumor clearance and triggering immune responses, potentially uncovering new therapeutic strategies that exploit the interplay between ferroptosis and immunity in cancer and other diseases.

Ferroptosis inducers have demonstrated potent cytotoxic effects against tumor cells in vitro, highlighting their potential as anticancer agents [67]. However, their therapeutic efficacy in vivo has been limited, potentially due to the tumor microenvironment's adaptive mechanisms, including antioxidant responses and DNA damage repair pathways that counteract ferroptotic stress. Our study identifies the DNA damage repair enzyme OGG1 (8‐oxoguanine DNA glycosylase 1) as a key player in tumor resistance to ferroptosis and shows that inhibiting OGG1 can synergistically enhance the cytotoxic effects of ferroptosis inducers.

OGG1 plays a central role in repairing oxidative DNA lesions [99, 100], such as 8‐oxoguanine, which are exacerbated under ferroptotic conditions. By inhibiting OGG1, the accumulation of DNA damage is amplified, increasing the vulnerability of tumor cells to ferroptosis‐induced lipid peroxidation and cell death. This combinatory approach leverages the interplay between oxidative stress and DNA repair pathways, providing a two‐pronged attack against tumor cells. Importantly, OGG1‐knockout mice have been shown to develop normally without significant physiological defects [101], suggesting that targeting OGG1 might represent a relatively safe therapeutic strategy with minimal off‐target effects.

Our findings suggest that targeting DNA damage repair pathways, particularly OGG1, can overcome the limitations of ferroptosis inducers in vivo and enhance their therapeutic efficacy. This strategy not only sensitizes tumor cells to ferroptosis but also may influence the tumor microenvironment by exacerbating oxidative stress. Future studies are needed to evaluate the safety and specificity of OGG1 inhibitors in combination with ferroptosis inducers, as well as to explore their effects on the immune system and tumor stroma. The apparent safety of OGG1 inhibition, combined with its efficacy in enhancing ferroptosis, provides a promising framework for developing safer and more effective combination therapies to harness ferroptosis for cancer treatment.

## 4 Methods

### 4.1 Chemicals and Reagents

RSL3 (T3646), erastin (T1765), ML210 (T8375), FINO2 (T60084), CsA (T0945), DFO (T124358), BSO (T5471), TH5487 (T8119), SU0268 (T9119), Fer‐1 (T6500), VBIT‐4 (T13287), H‐151 (T5674) and RU.521 (T5486) were ordered from TargetMol; Arachidonic acid (HY‐109590), Ardenic acid (HY‐W013215), and NIM811 (HY‐P0025) was purchased from MedChemExpress; IKE (MC2024) was purchased from MeilunBio. All chemicals were prepared as stock solutions in DMSO. Stock solutions were stored at −20°C.

### 4.2 Cell Lines and Cell Culture

NC1‐H1299 (CRL‐5803), U937 (CRL‐1593.2), 293T (CRL‐3216), and HK‐2 (CRL‐2190) cell lines were from the American Type Culture Collection. MEF cell line was isolated and immortalized from C57BL/6 wild‐type mice at 13.5–14.5 days of gestation in this laboratory. NCI‐H1299 cells, 293T cells, and MEF cells were cultured in Dulbecco's Modified Eagle's Medium supplemented with 10% FBS (Sigma–Aldrich), U937 cells were cultured in RPMI‐1640 medium supplemented with 10% FBS (Sigma–Aldrich). HK‐2 cells were cultured in F‐12 medium supplemented with 10% FBS (Sigma‐Aldrich). All cell lines were housed within a thermo tissue culture incubator (37°C, 5% CO_2_).

### 4.3 Cell Survival Assay

For the cell survival assay, we utilized the CellTiter‐Glo Luminescent Cell Viability Assay kit. In brief, cells (1 × 10^4^ MEF cells, 3 × 10^3^ NCI‐H1299 cells, 3 × 10^3^ HK‐2 cells, 1 × 10^4^ U937 cells) were plated in a 96‐well plate (Jet Biofil) and allowed to incubate for 24 h at 37°C with 5% CO_2_ before the designated treatment. Various concentrations of the compounds were added to the culture medium with a final 0.1% DMSO. The CellTiter‐Glo Assay (Promega) was conducted following the manufacturer's guidelines. Luminescence measurements were acquired using the PerkinElmer plate reader.

### 4.4 Analysis of General Lipid Peroxidation and Mitochondrial Lipid Peroxidation

C11‐BODIPY (581/591) from Molecular Probes and MitoPerOx from Cayman Chemical were employed to detect general lipid peroxidation and mitochondrial lipid peroxidation, respectively, following the manufacturers' instructions. The day before the experiment, 2 × 10^5^ NCI‐H1299 cells were seeded in 6‐well plates (Jet Biofil). On the day of the experiment, cells were treated with the indicated compounds for the specified durations. The cells were then collected through trypsinization and centrifugation, washed with PBS, and suspended in 200 µL of Opti‐MEM (Gibco) containing either C11‐BODIPY (581/591) (5 µm) or MitoPerOx (100 nm). After a 30 min incubation at room temperature in the dark, the Opti‐MEM was removed by centrifugation, and the cells were resuspended in 200 µL of PBS. Subsequent analysis was conducted using a CytoFlex flow cytometer (Beckman Coulter). Data were collected from the FL1 channel corresponding to C11‐BODIPY/MitoPerOx. A minimum of 20 000 cells were analyzed per condition. FACS analysis was carried out using FlowJo V10 software.

### 4.5 Western Blot

For cell samples, Western blot samples were prepared by directly adding 4x SDS loading buffer (comprising 100 mm Tris‐HCl (T5941, Sigma‐Aldrich), pH = 6.8, 8% β‐mercaptoethanol, 10 mm DTT, 7.32 mm SDS, 20% Glycerol, 2% Bromophenol blue (B802654, Macklin)) to the cell pellet. Protein lysates were loaded onto SDS‐PAGE for further analysis.

Immunoblot analysis was conducted using antibodies that specific to GPX4 (1:10000; cat. no. ab231174, Abcam), DHODH (1: 4000; cat. no.14877‐1‐AP, Proteintech), TFRC (1: 2000, cat. no. A5865, Abclonal), ACSL4 (1: 200; cat. no. sc‐271800, Santa Cruz Biotechnology), CypD (1:4000, cat. no. 18466‐1‐AP, Proteintech), FSP1 (1:1000, cat. no. 20886‐1‐AP, Proteintech), ANT2 (1:2000, cat. no. 14841‐1‐AP, Proteintech), SPG7 (1:2000, cat. no. A10249, Abclonal), AFG3L2(1:4000, cat. no.14631‐1‐AP, Proteintec), TBK1 (1:1000, cat. no. 38066S, Cell Signaling Technology), p‐TBK (1:1000, cat. no. 5483T, Cell Signaling Technology), c‐GAS (1:1000, cat. no. 15102S, Cell Signaling Technology), STING (1:1000, cat. no. 13647S, Cell Signaling Technology), Tomm 20 (1:2000, cat. no. sc‐17764, Santa Cruz Biotechnology), FTH1 (1:1000, cat. no. 14768‐1‐AP, Proteintech), FEN1 (1:1000, sc‐28355, Santa Cruz Biotechnology), GAPDH (1:100000, 60004‐1‐lg, Proteintech) and α‐Tubulin (1:10000; cat. no. 66031‐1‐lg, Proteintech).

### 4.6 Immunofluorescence Microscopy

NCI‐H1299 cells were seeded at a density of 1 × 10^4^ cells per well in 3.5 cm glass‐bottom dishes. The cells were then treated with the indicated ferroptosis inducers. Mitochondrial pore opening was assessed using the calcein–CoCl_2_ method [102]. Briefly, treated H1299 cells were incubated with 1 µm calcein–AM (Beyotime, C2009S) plus 1 mm CoCl_2_ in HBSS for 15 min at 37°C. Calcein‐loaded cells were then washed twice with HBSS, and fluorescence images were collected using a laser scanning confocal microscope (Olympus IX83).

For mitochondrial DNA detection, the cells were incubated with 100 nm MitoTracker (40741ES50, Yeasen Biotechnology) and PicoGreen for 30 min, followed by three washes with PBS. Images were acquired using an HIS‐SIM microscope equipped with a 100× objective lens. 3D surface reconstruction and high‐magnification snapshots of selected regions were performed using Imaris software (v9.12).

### 4.7 Determination of GSH Levels

NCI‐H1299 control and CypD KO cells were seeded at a density of 3000 cells per well into a 96‐well plate (Jet Biofil). Following overnight incubation, the cells were treated with the indicated concentration of erastin or BSO for 18 h. After removal of the medium, GSH levels were assessed following the GSH‐Glo Glutathione Assay Protocol (Promega). The quantification of GSH levels was based on luminescence measurements using the Perkin Elmer plate reader as counts per second with an integration time of 1 s. The average of triplicate data points was calculated and normalized to the DMSO control.

### 4.8 CRISPR‐Cas9 Knockout Cell Line Generation

The specific sgRNAs utilized for gene knockout of interest and the non‐targeting control sgRNA were as follows:

Human CypD sgRNA
1#: 5’‐TACCTGGACGTGGACGCCAA‐3’
2 #: 5’‐AACTTCAGAGCCCTGTGCAC‐3’
3 #: 5’‐AGCCCTTCTCACCAGTGCAC‐3’
None targeting control sgRNA (5’‐GAAATGCTATGCTTCGGTTC‐3’).

These sequences were inserted into lentiCRISPR‐v2 (Addgene #52961) vectors. High‐titer lentiviral particles were generated using 293T cells. The virus‐containing medium was collected 48 h post‐transfection, and transduction was carried out once cell confluence exceeded 80%. Selection was achieved using puromycin (1 µg/mL). The knockout efficiency of CRISPR‐Cas9 was validated via western blot analysis.

### 4.9 siRNA Transfection

The following siRNAs were employed for the knockdown of target genes:

Human ANT1 siRNAs:
1#: 5’‐AGUACAAAGGGAUCAUUGATT‐3’,
2#: 5’‐ACAGAUCAGUGCUGAGAAGdTdT‐3’
Human ANT2 siRNAs:
1#: 5’‐GCUCUACUUUGCAGGGAAUTT‐3’,
2#: 5’‐GCCUGUACCAAGGCUUUAATT‐3’
Human ANT3 siRNAs:
1# 5’‐GGAGCUGACAUCAUGUACATT ‐3’,
2# 5’‐GAGCUCAAGAAGGUGAUCUTT ‐3’
Human ANT4 siRNAs:
5’‐AUUCAUGUCUGGAGUUAAUTT ‐3’
Human ATP5PB siRNAs:
1# 5’‐GGGUGGUACUUUCCGCCGCTT‐3’,
2# 5’‐AGCGCAGAGACCUUCACUGTT‐3’,
3# 5’‐ACGGAGAAGUCACAACAGGTT‐3’
Human ATP5MC1 siRNAs:
1# 5’‐GACCGCCGGGGCAUUAUUCTT‐3’,
2# 5’‐AGCCCAGUGAAUUCAUCUATT‐3’,
3# 5’‐GGCUGGCAUUGGAACCGUGTT‐3’
Human SPG7 siRNAs:
1#: 5’‐GCUCUGCAGAGCUUACAAUTT‐3’,
2#:5’‐GGUUGAAGCAGAAGAAUAATT‐3’
Human AFG3L2 siRNAs:
1#: 5’‐GGCAAUACGUUUGGUUUAATT‐3’,
2#: 5’‐GGUCGAGUCUCUGAAGAAATT‐3’
Human FEN1 siRNAs:
1#: 5’‐GCCCGUGUAUGUCUUUGAUTT‐3’,
2#: 5’‐GGGCAUCCCUUAUCUUGAUTT‐3’
Human cGAS siRNAs:
1#: 5’‐CGUGAAGAUUUCUGCACCU‐3’
2#: 5’‐GCAAAAGUUAGGAAGCAAC‐3’
Non‐targeting siRNA: 5’‐UUCUCCGAACGUGUCACGUTT‐3’ as a negative control.

All siRNAs were synthesized by Shanghai GenePharma Co., Ltd (Shanghai, China) and transfected using Lipofectamine RNAiMAX (Invitrogen) as per the manufacturer's guidelines. The efficiency of siRNA knockdown was verified through western blot analysis.

### 4.10 RNA Isolation and Quantitative Real‐time PCR (qPCR)

RNA was isolated using the EZ‐press RNA Purification Kit (B0004DP), and cDNA synthesis was performed using the Evo M‐MLV transcription premix kit (AG11728). mRNA expression levels were assessed via QPCR carried out in a CFX96 thermal cycler (Bio‐Rad) following established protocols. The data are represented in arbitrary units and quantified using the 2^(‐ΔΔCT) method. Primer sequences were as follows:

ND1 Forward: CACCCAAGAACAGGGTTTG,
ND1 Reverse: TGGCCATGGGTATGTTGTTAA,
29 18S Forward: TAGAGGGACAAGTGGCGTTC,
30 18S Reverse: CGCTGAGCCAGTCAGTGT,
Actin Forward: CATGTACGTTGCTATCCAGGC,
Actin Reverse: CTCCTTAATGTCACGCACGAT,
ANT1 Forward #1: ATCACGCTTGGAGCTTCCTAA,
ANT1 Reverse #1: TGCTTCTCAGCACTGATCTGT,
ANT1 Forward #2: CTCTCCTTCTGGAGGGGTAAC,
ANT1 Reverse #2: GAACTGCTTATGCCGATCCAC,
ANT2 Forward #1: TTATAGACTGCGTGGTCCGTA,
ANT2 Reverse #1: GGCGAAGTTAAGAGCCTGGG,
ANT2 Forward #2: AGTTTTGGCTCTACTTTGCAGG,
ANT2 Reverse #2: GGCCCTTAATCCCATCAGATTTG,
ANT3 Forward #1: GCAACCTTGCCAACGTCATTC,
ANT3 Reverse #1: CCGCAAAGTACCTCCAGAACT,
ANT3 Forward #2: CAGCGGACGTGGGAAAGTC,
ANT3 Reverse #2: TTGGCCGTATCGTACACGC.
ANT4 Forward #1: CATCGTGAGCCTGCGAAAAAG
ANT4 Reverse #1: GGGCTGATCTGCTTCGACG
ANT4 Forward #2: CGCGGTACAAAGGCATGGT
ANT4 Reverse #2: ATTGCCACGCCAAAAACTGAA
ATP5PB Forward: AGGTCCAGGGGTATTGCAG
ANT5PB Reverse: TCCTCAGGGATCAGTCCATAAC
ATP5MC1 Forward: CTGTTGTACCAGGGGTCTAATCA
ANT5MC1 Reverse: GTGGGAAGTTGCTGTAGGAAG

### 4.11 Cellular Fractionation and Measurement of Cytosolic mtDNA

NCI‐H1299 cells were treated as specified. In brief, NCI‐H1299 cells were rinsed with PBS, harvested, with 10% saved for whole‐cell DNA extraction. The remaining cells were resuspended in pre‐chilled mitochondrial extraction buffer 1 (comprising 220 mm mannitol, 70 mm sucrose, 20 mm HEPES‐KOH, pH 7.5, 1 mm EDTA, and 2 mg/mL bovine serum albumin) supplemented with a protease inhibitor cocktail (K1007, Apexbio) and a phosphatase inhibitor cocktail (K1014, Apexbio). After incubation on ice for 20 min, the cells were passed through a 25‐G syringe (BD Biosciences) ten times on ice. The homogenized cells were then centrifuged at 1000 g for 15 min at 4°C. The resulting supernatant underwent further centrifugation at 10 000 g for 10 min at 4°C to isolate the mitochondria from the cytosolic fraction. After DNA purification using the AFastPure Cell/Tissue DNA Isolation Mini Kit (DC102, Vazyme) according to the manufacturer's instructions, qPCR was carried out on both whole cell extracts and cytosolic fractions using mtDNA primers (ND1). Additionally, qPCR was performed on whole cell extracts using nuclear DNA primers (18S) or actin. The Ct values obtained for nuclear DNA abundance in whole cell extracts were utilized as normalization controls for the mtDNA values obtained from the cytosolic fractions.

### 4.12 Elisa (Enzyme‐Linked Immunosorbent Assay)

For the measurement of Ox‐mtDNA, purified mtDNA was extracted from the cytosolic or mitochondrial fractions as indicated. The Ct values obtained for mtDNA abundance in whole cell extracts served as normalization controls for the Ox‐mtDNA values obtained from the cytosolic fractions. A set of paired antibodies (capture and detection) was employed to quantify 8‐OHdG concentrations following the manufacturer's guidelines (RK07128, Abclonal).

### 4.13 RNA‐Sequencing

Total RNA was extracted using the Trizol reagent kit (Invitrogen, Carlsbad, CA, USA) according to the manufacturer's protocol. RNA quality was assessed on an Agilent 2100 Bioanalyzer (Agilent Technologies, Palo Alto, CA, USA) and checked using RNase‐free agarose gel electrophoresis. After total RNA was extracted, eukaryotic mRNA was enriched by Oligo(dT) beads. Then the enriched mRNA was fragmented into short fragments using the fragmentation buffer and reverse transcribed into cDNA by using NEBNext Ultra RNA Library Prep Kit for Illumina (NEB #7530, New England Biolabs, Ipswich, MA, USA). The purified double‐stranded cDNA fragments were end‐repaired, A base added, and ligated to Illumina sequencing adapters. The ligation reaction was purified with the AMPure XP Beads(1.0X). And the polymerase chain reaction (PCR) amplified. The resulting cDNA library was sequenced using Illumina Novaseq X plus by Gene Denovo Biotechnology Co. (Guangzhou, China).

### 4.14 In Vivo Xenograft Studies

The ethical approval of the animal experiment was obtained from the Animal Care & Welfare Committee and the Department of Lab Animal Science of Shanghai Jiao Tong University School of Medicine (Approval number: JUMC2023‐015‐A). NCI‐H1299 xenografts were established in 6‐week old BALC nu/nu mice by injecting 5 × 10^6^ cells into the right back of the mice subcutaneously. Once tumor volume reached ≥50 mm^3^, mice were divided randomly into 4 groups (n = 7): the vehicle, IKE (40 mg/kg) ‐only, TH5487 (30 mg/kg)‐only, and IKE + TH5487 combination group. All the treatments were injected intratumorally; these chemicals were administered every day. Tumors were measured every day by calipers. 8 days after administration, the mice were euthanized, and tumor were dissected and weighed.

### 4.15 Statistical Analyses

Data are presented as mean ± standard deviation (s.d.) unless otherwise specified. Statistical comparisons among various groups were analyzed using One‐way ANOVA (Prism; GraphPad). The intra‐group comparison used a two‐way ANOVA.

## Author Contributions

H.Z. and Z.Z. started the project and H.Z. performed most of the experiments; W.F. finalized the project revision with the help of H.Z. and S.L.; H.L. helped with the animal experiments. H.Z., W.F., and Q.Z. conceived the project, designed the experiments, analyzed the data, and wrote the manuscript. All authors discussed the results and commented on the manuscript.

## Conflicts of Interest

The authors declare no conflict of interest.

## Supporting information




**Supporting File**: advs74193‐sup‐0001‐SuppMat.docx.

## Data Availability

The raw RNA‐seq data generated in this study have been deposited in the NCBI Sequence Read Archive (SRA) under accession number PRJNA1414070. The data that support the findings of this study are available in the supplementary material of this article.
