# Supplementary material for: CypD Dependent mPTP Opening Is Crucial for Oxidized Mitochondrial DNA Release in Ferroptosis
Source: Adv Sci (Weinh). 2026 Feb 17;13(20):e02239. doi: 10.1002/advs.202502239 (PMC13067831; doi:10.1002/advs.202502239)
Supplement: Supplementary file 1 — Supporting File: advs74193‐sup‐0001‐SuppMat.docx. [file ADVS-13-e02239-s001.docx]

Supporting Information

**CypD dependent mPTP opening is crucial for oxidized mitochondrial DNA release in ferroptosis**

*Hong Zhou^1,#^, Wan Fu^1,#,*^, Shizuo Liu^1^, Zili Zhang^1^, Hanyan Luo^1^, Qing Zhong^1,*^*


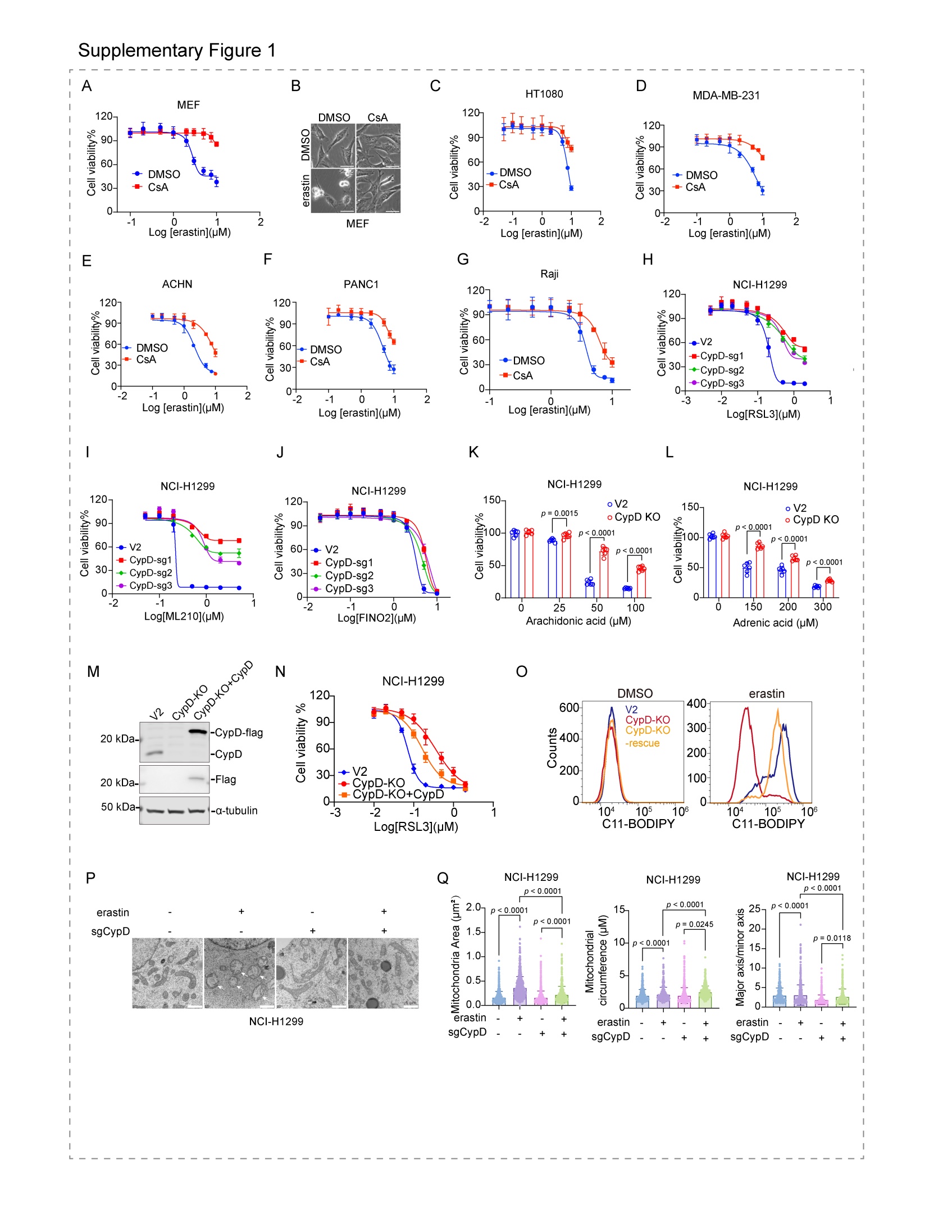
**Figure S1. CypD is essential for the execution of ferroptosis.**

**A.** MEF cells were treated with increased doses of erastin as indicated in the presence of CsA (5 μM) for 24 hours, then cell survival rate was measured by Cell Titer Glo Viability Assay. **B.** The effect of CsA on morphology of MEF cells treated with erastin (5 μM) for 24 hours. Scale bar: 50 µm. **C-G.** HT1080 (**C**), MB-MDA-231 (**D**), ACHN (**E**), PANC1 (**F**), Raji (**G**) cells were treated with increased doses of erastin as indicated in the presence of CsA (5 μM) for 24 hours, cell survival rate was measured by Cell Titer Glo viability assay. **H.** Cell death of NCI-H1299 WT and CypD KO cells subjected to indicated doses of RSL3 for 6 hours. Cell viability was measured by Cell Titer Glo assay. **I-J**. Cell death of NCI-H1299 WT and CypD KO cells subjected to indicated doses of ML210 for 8 hours (**I**) and FINO2 for 24 hours (**J**) respectively. Cell viability was measured by Cell Titer Glo assay. **K-L**. Cell death of NCI-H1299 WT, CypD KO cells subjected to indicated doses of arachidonic acid (**K**), adrenic acid (**L**). **M.** Western blot analysis of CypD expression in NCI-H1299 control, CypD knockout and CypD KO supplemented with CypD cells. **N.** Cell death of NCI-H1299 WT, CypD KO and CypD KO supplemented with CypD cells subjected to indicated doses of RSL3 for 8 hours. Scale bar: 50 µm. **O.** NCI-H1299 control, CypD knockout and CypD KO supplemented with CypD cells were subjected to 5 μM erastin for 18 hours. Lipid peroxidation was measured with C11-BODIPY staining by flow cytometry. **P.** Transmission electron microscopy analysis of mitochondria in NCI-H1299 WT and CypD KO cells treated with or without RSL3. Scale bar: 1 µm. **Q.** Quantitative analysis of mitochondria area (left), mitochondrial circumference (middle) and the ratio between major axis and minor axis (right) in (**P**), ROI = 60, 51, 64, 50 respectively. The statistical significance in (**Q**) between different groups was analyzed by One-way ANOVA (Prism; GraphPad). Two-Way ANOVA were used for **K, L**.


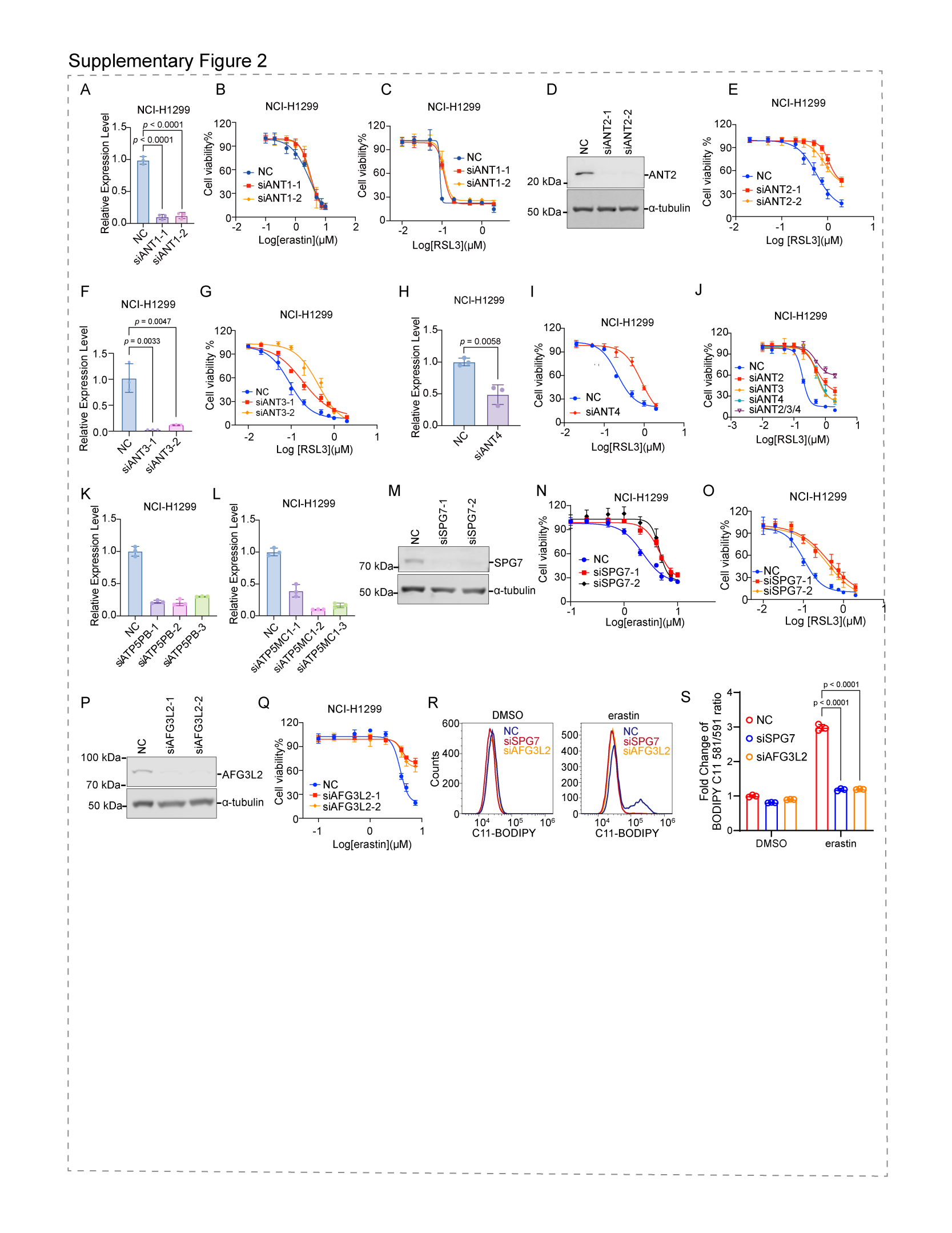


**Figure S2. mPTP machinery is involved in ferroptosis.**

**A.** qPCR analysis for ANT1 expression level. **B.** ANT1 was knocked down by specific siRNA in NCI-H1299 cells, followed by indicated doses of erastin for 24 hours (**B**) or RSL3 for 8 hours (**C**) to detect cell viability. **D.** Western blot analysis of ANT2 expression in NCI-H1299 control or knockdown cells. **E.** ANT2 was knocked down by specific siRNA in NCI-H1299 cells, followed by indicated doses of RSL3 for 8 hours to detect cell viability. **F.** qPCR analysis for ANT3 expression level. **G.** ANT3 was knocked down by specific siRNA in NCI-H1299 cells, followed by indicated doses of RSL3 for 8 hours to detect cell viability. **H.** qPCR analysis for ANT4 expression level. **I.** ANT4 was knocked down by specific siRNA in NCI-H1299 cells, followed by indicated doses of RSL3 for 8 hours to detect cell viability. **J**. ANT2, ANT3, and ANT4 were knocked down simultaneously by specific siRNA in NCI-H1299 cells，followed by indicated doses of RSL3 for 8 hours to detect cell viability. **K, L.** QPCR analysis of expression level of ATP5PB (**K**), ATP5MC1 (**L**) in indicated control and knockdown cells. **M, P.** Western blot analysis of SPG7 (**M**) and AFG3L2 (**P**) expression in indicated cells. **N, O, Q.** NCI-H1299 cells were transfected with siRNA respectively against SPG7 (**N-O**), and AFG3L2 (**Q**) for 48 hours, followed by indicated doses of erastin for 24 hours or RSL3 for 8 hours to detect cell viability. **R.** NCI-H1299 cells were transfected with siRNA specially against SPG7 or AFG3L2 for 48 hours, then subjected to 5 μM erastin for 18 hours. Lipid peroxidation was measured with C11-BODIPY staining by flow cytometry. **S.** Quantitative analysis of the fold change of lipid oxidation ratio in (**R**)**.** The statistical significance between different groups (**A**, **F** and **H**) was analyzed by One-way ANOVA (Prism; GraphPad). Two-Way ANOVA was used for **S**.


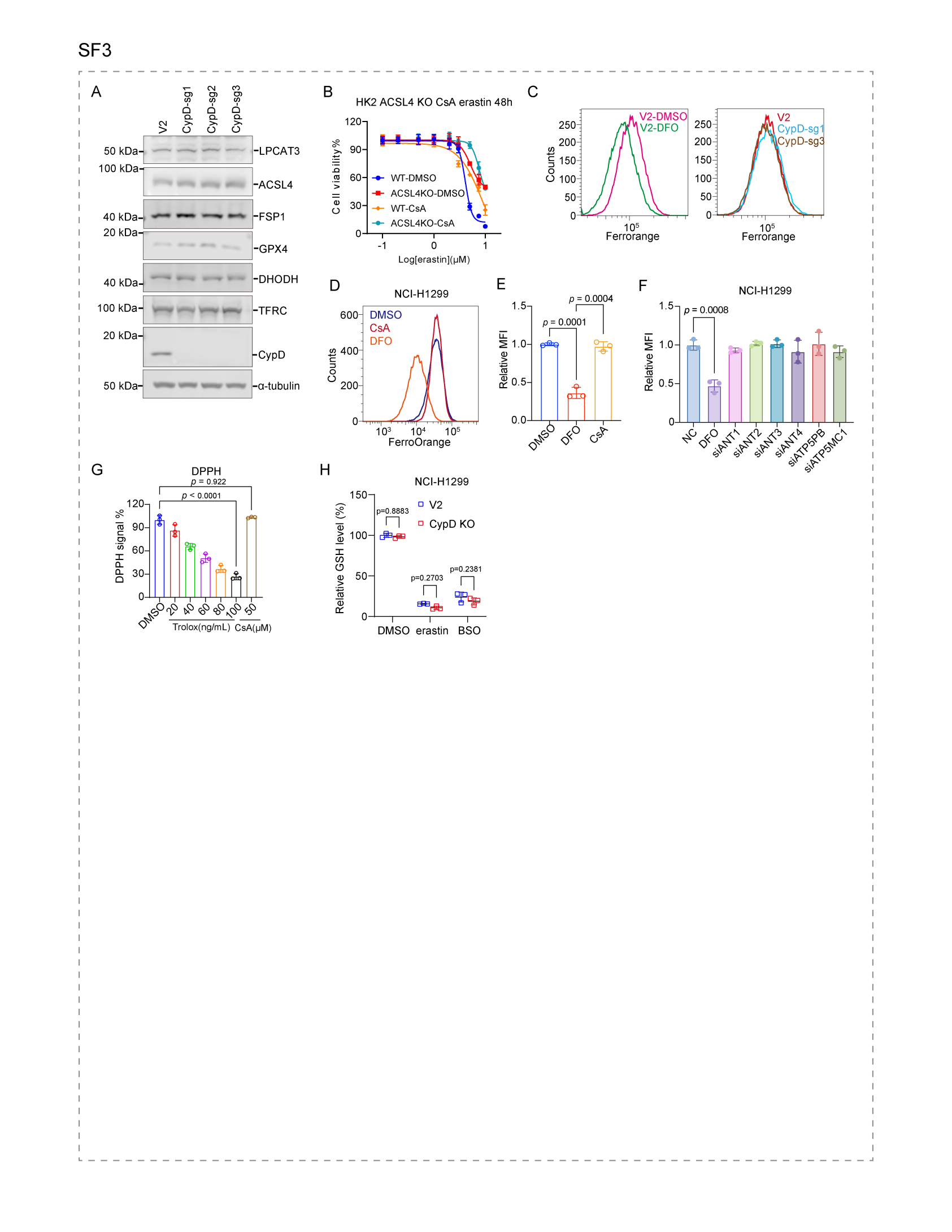
**Figure S3. mPTP does not directly interfere with iron metabolism or anti-oxidant production**

**A.** Immunoblotting for Lpcat3, ACSL4, FSP1, GPX4, DHODH, and TFRC protein levels in NCI-H1299 control and CypD KO cells. **B**. Cell viability analysis in HK-2 WT or ACSL4^-/-^ cells incubated with or without erastin for 48 hours following DMSO, Cyclosporine A (5 μM) treatment. **C.** NCI-H1299 control or CypD KO cells were labeled with FerroOrange to detect the cellular labile iron pool by flow cytometry. NCI-H1299 cells treated with 100 μM DFO were used as positive control. **D.** NCI-H1299 cells treated with indicated inhibitors were labeled with FerroOrange to detect the cellular labile iron pool by flow cytometry. **E.** Quantitative analysis of mean fluorescence intensity for (**D**). **F.** Indicated mPTP component was respectively knocked down by specific siRNA in NCI-H1299 cells for 48 h, then all the cells were labeled with FerroOrange to detect the cellular labile iron pool by flow cytometry. NC siRNA transfected cells treated with 100 μM DFO were used as positive control. Relative mean fluorescence intensities were shown in the histogram. **G**. The capacity of antioxidant activity of Cyclosporine A in DPPH assay. Trolox worked as positive control. **H.** GSH abundance was measured using GSH-Glo™ Glutathione Assay in NCI-H1299 control and CypD KO cells treated with indicated concentrations of erastin (5 μM) for 18 hours or BSO (200 μM). The statistical significance inside groups (**E** and **F**) was analyzed by One-way ANOVA (Prism; GraphPad). Two-Way ANOVA was used for (**H**) (Prism; GraphPad).


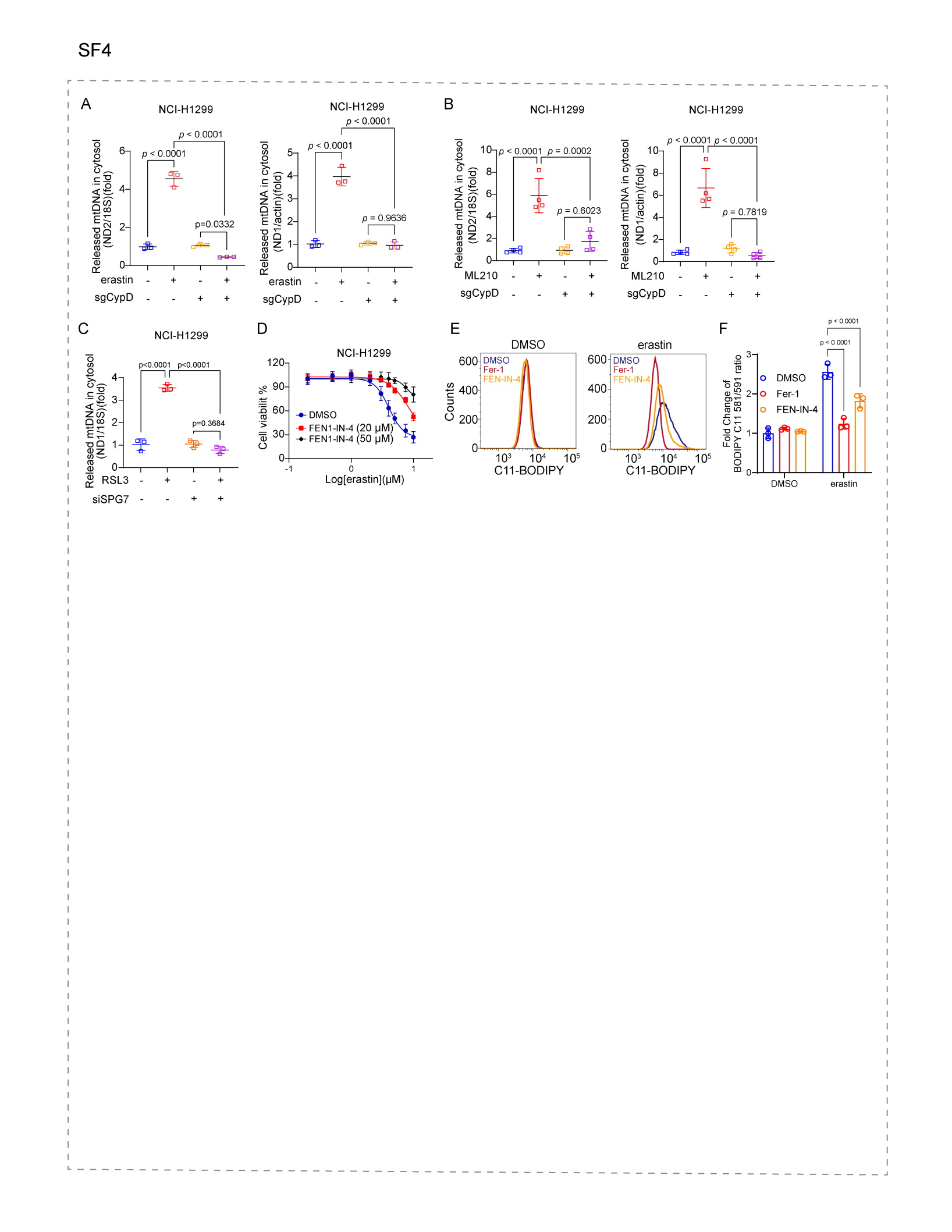
**Figure S4. MtDNA release mediated by mPTP promotes ferroptosis.**

**A.** Relative cytosolic mtDNA amounts in erastin treated NCI-H1299 WT and CypD-KO cells. The relative ratios of ND1 mtDNA, 18S nuclear DNA (**left panel**) and actin (**right panel**) are shown. **B.** Relative cytosolic mtDNA amounts in ML210 treated NCI-H1299 WT and CypD-KO cells. The relative ratios of ND1 mtDNA, 18S nuclear DNA (**left panel**) and actin (**right panel**) are shown. **C**. SPG7 was knocked down by specific siRNA in NCI-H1299 cells, followed by RSL3 treatment for 6 hours to detect relative cytosolic mtDNA. **D.** NCI-H1299 were treated with increased doses of erastin as indicated in the presence of FEN1-IN-4 (20, 50 μM) for 24 hours, then cell survival rate was measured by Cell Titer Glo Viability Assay. **E.** Lipid peroxidation level in NCI-H1299 cells with erastin treatment for 18 hours in the presence of DMSO, ferrostatin-1 (Fer-1, 2 μM) or FEN1-IN-4 (50 μM). The lipid peroxidation level was determined using C11-BODIPY staining by flow cytometry. Ferrostatin-1 (Fer-1) was used as a positive control. **F.** Quantitative analysis of the fold change of lipid oxidation ratio in (**E**)**.** The statistical significance between different groups (**A-C**) was analyzed by One-way ANOVA (Prism; GraphPad). Two-Way ANOVA were used for **F**.


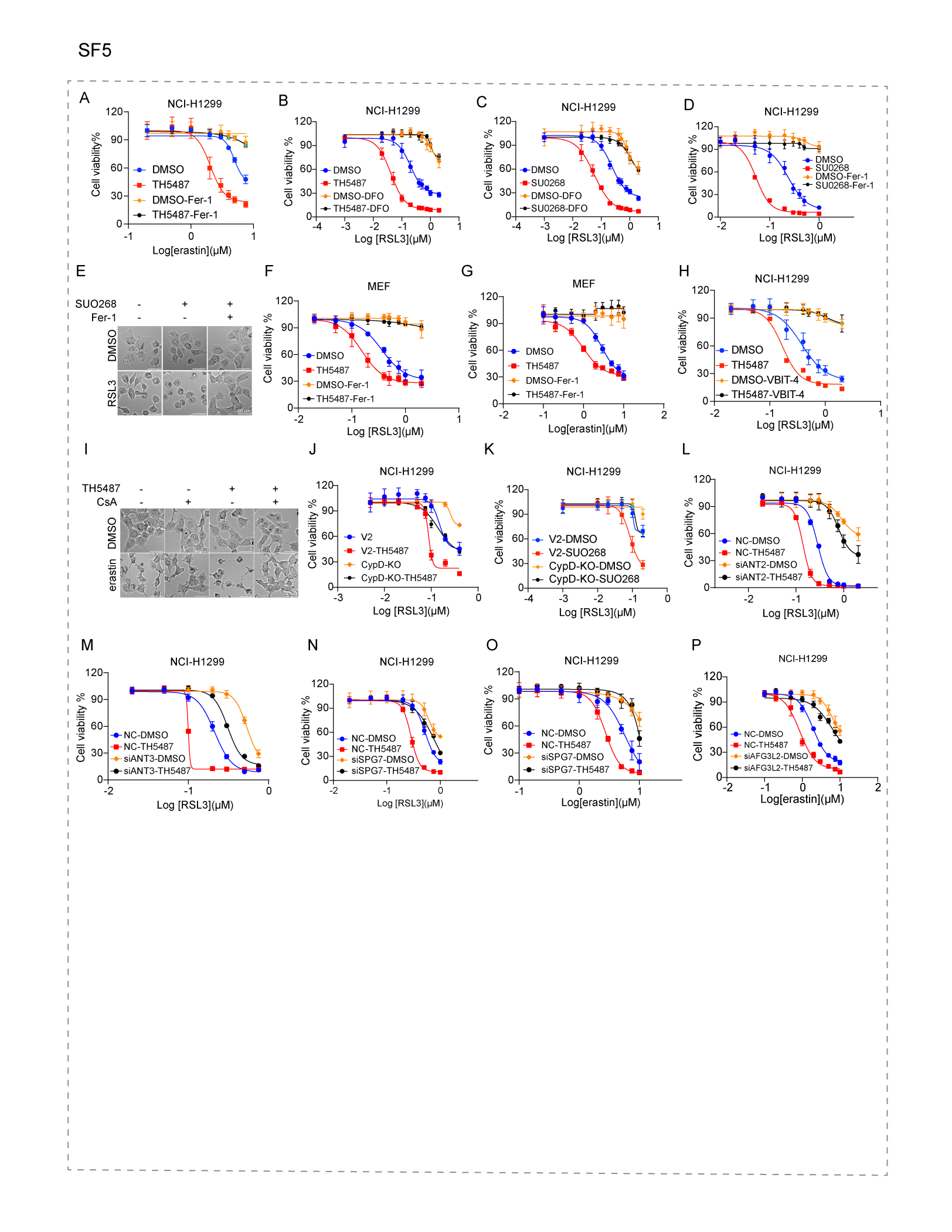
**Figure S5. OGG1 inhibitors promotes ferroptosis via an mPTP-dependent mechanism.**

**A**. NCI-H1299 were treated with increased doses of erastin as indicated in the presence of TH5487 (5 μM) and Fer-1 (2 μM) for 24 hours; then cell survival rate was measured by Cell Titer Glo Viability Assay. **B-C**. NCI-H1299 were treated with increased doses of RSL3 (**C**) or erastin (**D**) as indicated in the presence of TH5487 (5 μM) and DFO (100 μM) for 8 hours or 24 hours respectively, then cell survival rate was measured by Cell Titer Glo Viability Assay. **D**. NCI-H1299 were treated with increased doses of RSL3 as indicated in the presence of SUO268 (5 μM) and Fer-1 (2 μM) for 8 hours; then cell survival rate was measured by Cell Titer Glo Viability Assay. **E.** The morphology of NCI-H1299 cells treated with RSL3 (0.5 μM) for 4 hours following SUO268 (5 μM) and Fer-1 (2 μM) pre-treatment. Scale bar was 50 µm. **F-G**. MEF cells were treated with increased doses of RSL3 (**F**) or erastin (**G**) as indicated in the presence of TH5487 (5 μM) and Fer-1 (2 μM) for 24 hours; then cell survival rate was measured by Cell Titer Glo Viability Assay. **H**. NCI-H1299 were treated with increased doses of RSL3 as indicated in the presence of TH5487 (5 μM), with or without VBIT-4 (5 μM) pretreatment for 8 hours, then cell survival rate was measured by Cell Titer Glo Viability Assay. **I**. The morphology of NCI-H1299 cells treated with erastin (5 μM) for 18 hours following TH5487 (5 μM) and CsA (5 μM) pre-treatment. Scale bar was 50 µm. **J-K**. CypD were knocked out in NCI-H1299 cells, then cells were treated with increased doses of RSL3 with or without TH5487 (5 μM, **J**), SUO268 (5 μM, **K**) pretreatment. Cell death was measured by Cell Titer Glo Viability Assay. **L-O**. ANT2 (**L**), ANT3 (**M**), SPG7 (**N)** were knocked down by specific siRNA in NCI-H1299 cells, then cells were treated with increased doses of RSL3 with or without TH5487 (5 μM) pretreatment. Cell death was measured by Cell Titer Glo Viability Assay.


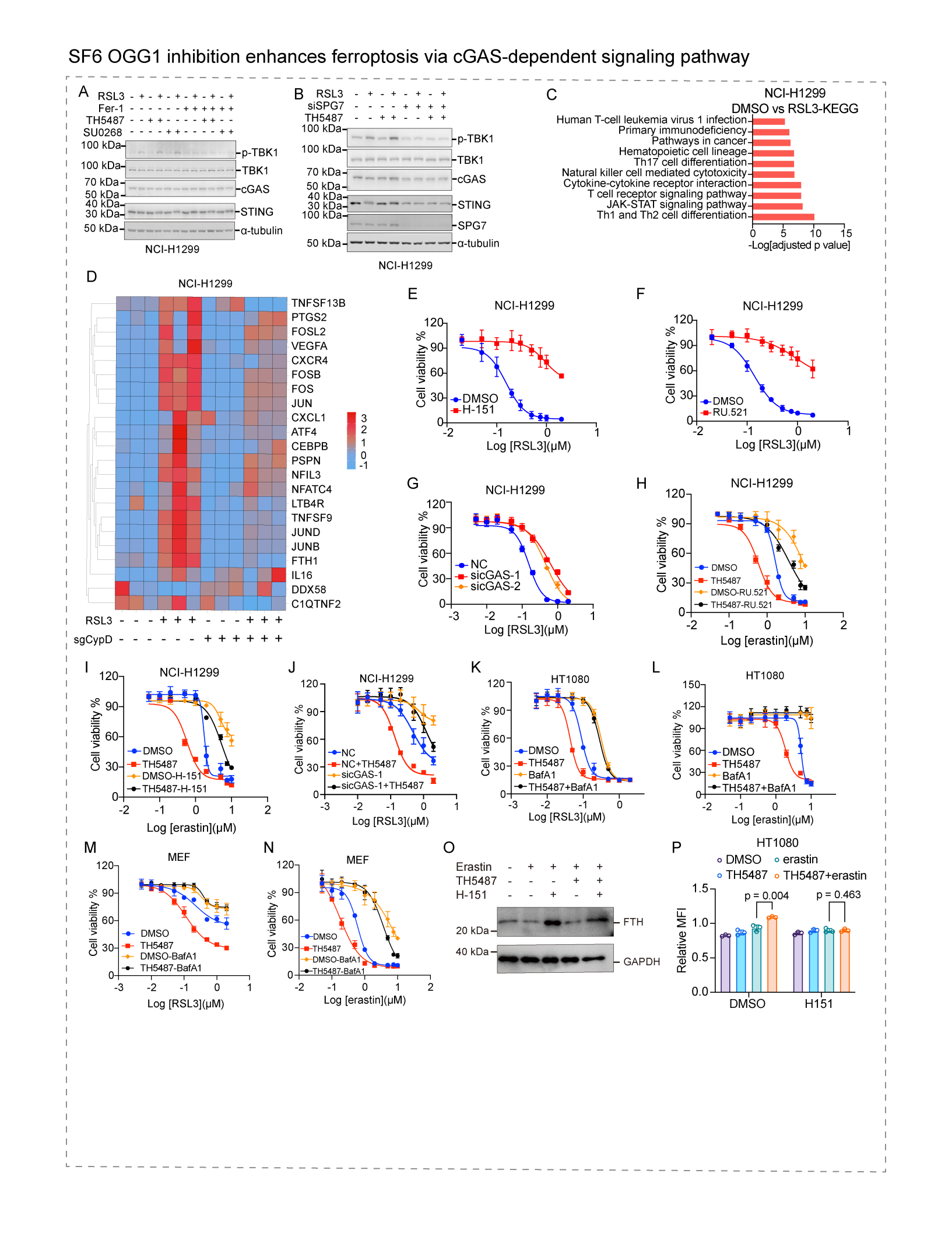
**Figure S6. OGG1 inhibitors exacerbate ferroptosis through cGAS-STING pathway.**

**A**. Western Blot analysis of TBK1 Ser172 phosphorylation and TBK1, STING, cGAS of RSL3-primed NCI-H1299 that were pretreated ± Fer-1 (2 μM), TH5487 (5 μM) or SU0268 (5 μM). **B.** SPG7 was knocked down, IB analysis of TBK1 Ser172 phosphorylation and TBK1, STING, cGAS of RSL3-primed NCI-H1299 that were pretreated ±TH5487 (5 μM). **C**. Significantly enriched KEGG pathways of the differential enriched genes. **D**. Heatmap showing differential immune-related feature counts for both NCI-H1299 WT and CypD knock out cell lines before and after RSL3 treatment sorted by logFC. **E-F**. NCI-H1299 were treated with increased doses of RSL3 as indicated with or without H-151 (2 μM, **E**) or RU.521 (10 μM, **F**) for 8 hours, then cell survival rate was measured by Cell Titer Glo Viability Assay. **G.** cGAS was knocked down by specific siRNA in NCI-H1299 cells, followed by indicated doses of RSL3 for 8 hours to detect cell viability. **H-I**. NCI-H1299 were treated with increased doses of erastin as indicated in the presence of TH5487 (5 μM) with or without H-151 (2 μM, **H**) or RU.521 (10 μM, **I**) for 24 hours, then cell survival rate was measured by Cell Titer Glo Viability Assay. **J**. cGAS was knocked down by specific siRNA in NCI-H1299 cells, then cells were treated with increased doses of RSL3 with or without TH5487 (5 μM) pretreatment. **K-L**. HT1080 were treated with increased doses of RSL3 (**K**) and erastin (**L**) as indicated in the presence of TH5487 (5 μM) and Bafilomycin A1 (BafA1, 100 nM) for 8 hours or 24 hours respectively, then cell survival rate was measured by Cell Titer Glo Viability Assay. **M-N.** MEF were treated with increased doses of RSL3 (**M**) and erastin (**N**) as indicated in the presence of TH5487 (5 μM) and Bafilomycin A1 (BafA1, 100 nM) for 6 hours or 24 hours respectively, then cell survival rate was measured by Cell Titer Glo Viability Assay. **O.** Western Blot analysis of FTH1 in NCI-H1299 cells treated with erastin in the presence of H-151 (3 μM) or TH5487 (5 μM). **P**. HT1080 cells were treated as indicated, then labile iron level was detected by FACS using FerroOrange. Relative mean fluorescence was shown. The statistical significance between different groups was analyzed by Two-way ANOVA (Prism; GraphPad).
